# Supplementary material for: Ronin Governs Early Heart Development by Controlling Core Gene Expression Programs
Source: Cell Rep. 2017 Nov 7;21(6):1562–73. doi: 10.1016/j.celrep.2017.10.036 (PMC5695914; doi:10.1016/j.celrep.2017.10.036)
Supplement: Document S1. Supplemental Experimental Procedures and Figures S1–S9 [file mmc1.pdf]

Cell Reports, Volume *21*

Supplemental Information

Ronin Governs Early Heart Development

by Controlling Core Gene Expression Programs

Jun Fujita, Pablo Freire, Cristian Coarfa, Ashley L. Benham, Preethi Gunaratne, Michael D. Schneider, Marion Dejosez, and Thomas P. Zwaka

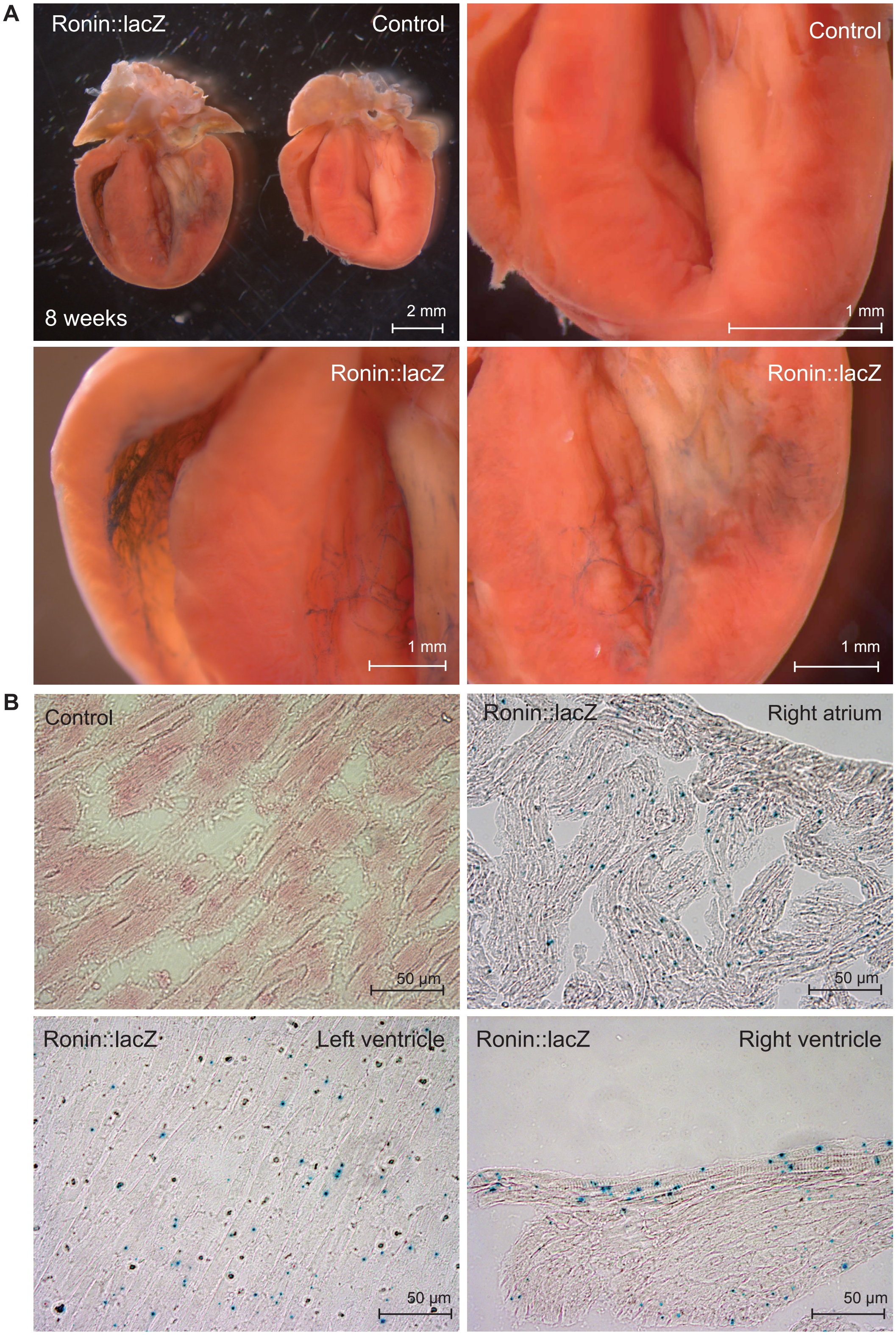

Figure S1: LacZ staining of *Ronin::lacZ* and control animals at 8 weeks of age. Related to Figure 1. (**A**) Images of entire hearts cut sagitally along the midsection. (**B**) Images of heart sections.

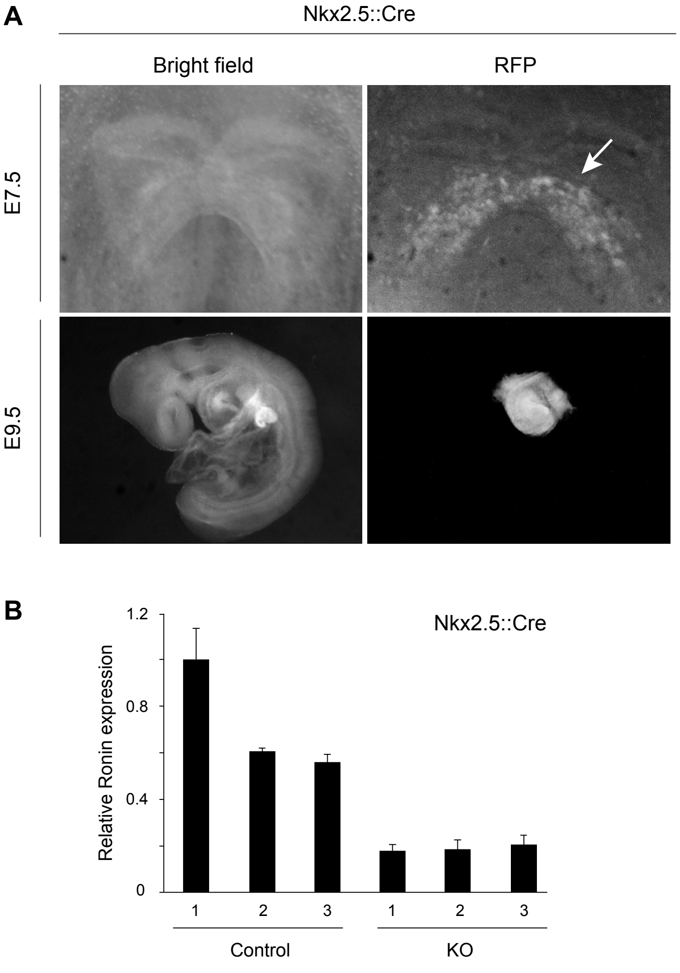

C

Ronin	Nkx2.5::Cre +/o	Nkx2.5::Cre o/o	αMHC::Cre +/o	αMHC::Cre o/o
fl/+	19	18	22	24
fl/fl	0	25	22	21

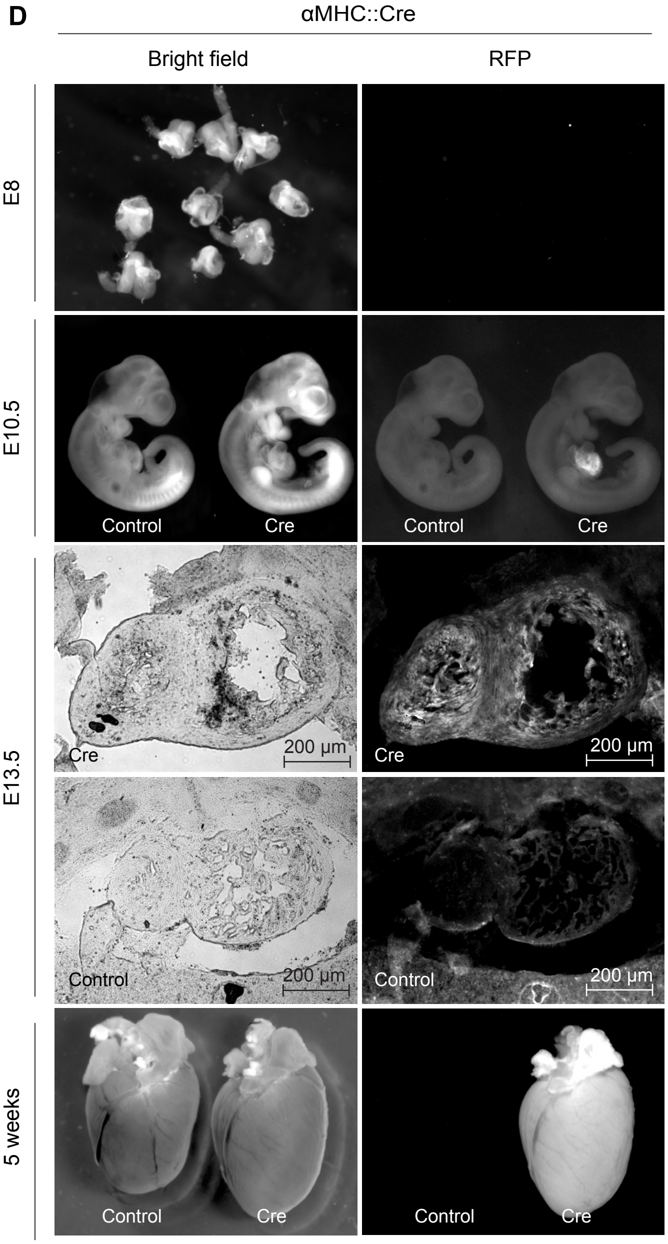

Figure S2. Validation of the temporal and spatial *Nkx.2.5*- and *αMHC*-driven Cre expression. Related to Figures 2 and 3. *Nkx2.5*::*Cre* or *αMHC*::*Cre* animals were crossed with *Rosa26*$^{\mathrm{tdRFP/tdRFP}}$ reporter animals, and RFP-positive cells were analyzed at various developmental stages. (**A**) *Nkx2.5::Cre*: RFP-positive cells emerge first in the cardiac crescent (white arrow) at E7.5, and then appear throughout the heart at E9.5. This supports the notion that *Nkx2.5*::Cre activity closely mirrors endogenous *Nkx2.5* expression and thus that the *Ronin* gene is excised at E7.5. (**B**) RT-PCR of *Ronin* at E11.5 confirmed significant reduction of Ronin expression after knockout (n=3; p=0.0187 by t-test). (**C**) Distribution of *Ronin* alleles in crosses with *Nkx2.5*::Cre and *αMHC*::Cre animals at P0 as indicated. No viable embryos were identified after *Nkx2.5*-driven knockout, whereas *αMHC*- knockout showed typical mendelian ratios. (**D**) *αMHC::Cre*: While no Cre activity is detected at E8.0, and from E10.5 on, the bulk of the heart is RFP-positive (>90% of all cardiac cells), suggesting *αMHC::Cre*-mediated conditional deletion of *Ronin* as early as E10.5 that is sustained thereafter, as seen in sections at E13.5 or at 5 weeks of age.

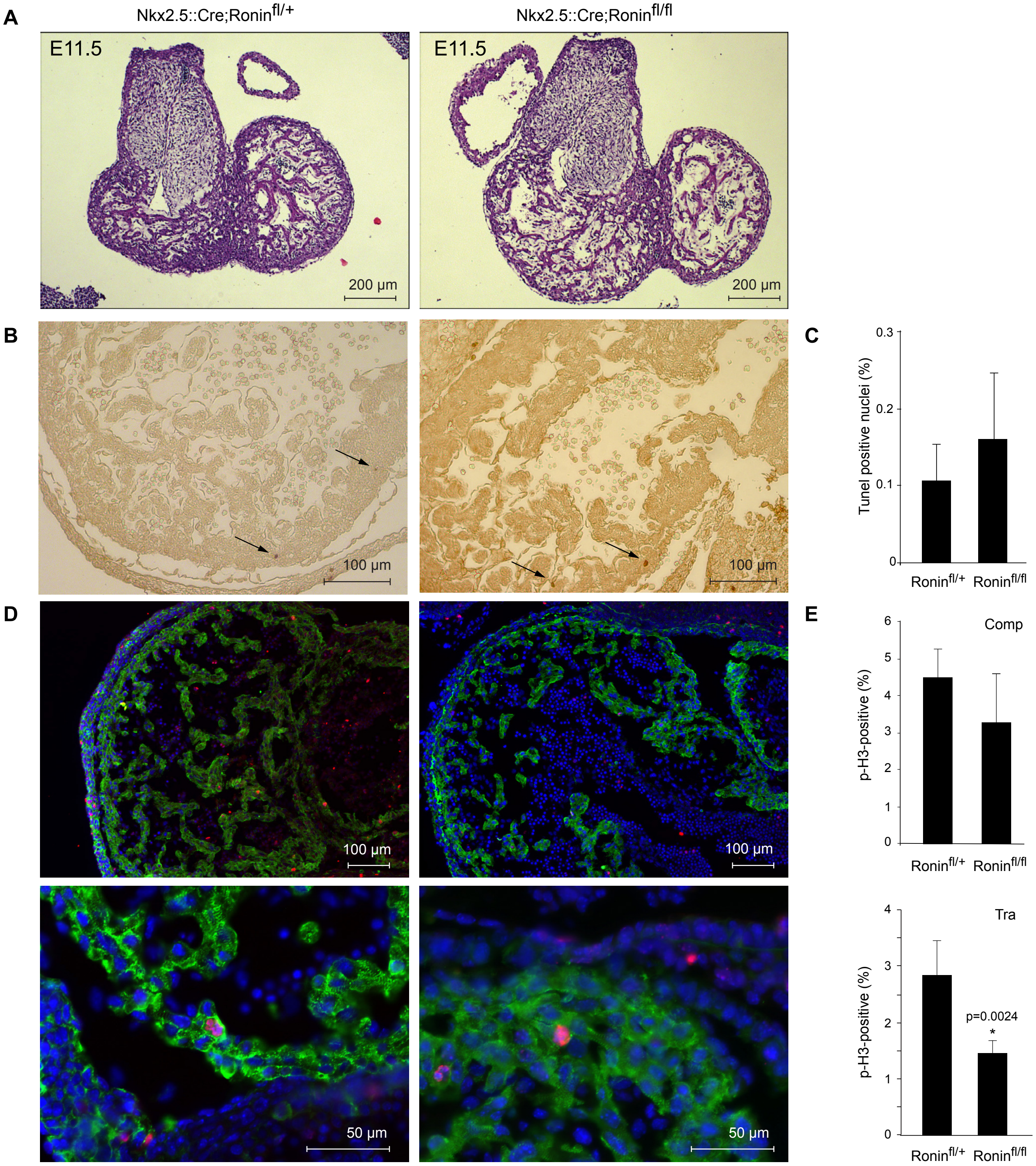

Figure S3. *Nkx2.5*-driven loss of Ronin leads to cardiac defects and is associated with cell death and attenuated cell proliferation by E11.5. Related to Figure 2. (**A**) Heart sections after H&E staining show that the heart chamber walls of *Nkx2.5*-driven *Ronin* knockout mice (right) are much thinner than those of heterozygous controls (left) at E11.5. (**B**) Sections of E11.5 heart tissue after tunel-staining to detect apoptotic cell death. (**C**) Quantification of experiment shown in (B) indicates an increase, albeit not statistically significant, in programmed cell death in *Nkx2.5*-driven *Ronin* knockout heart tissue. (**D**) Phospho-Histone 3 (p-H3) immunostaining of E11.5 heart tissue sections shows a decrease of mitotic cells in *Nkx2.5*-driven *Ronin* knockout animals when compared with heterozygous controls in the free ventricular walls (excluding atrium and septum). Red, p-H3; green, α–Actinin; blue, Dapi. (**E**) Quantification of experiment shown in (D) confirms a decrease of mitotic cells in both myocardial layers with a statistically significant difference in the trabecular layer. Comp, compact layer; Tra, trabecular layer. Five animals per group were analyzed and, at least 3600 nuclei were counted per layer.

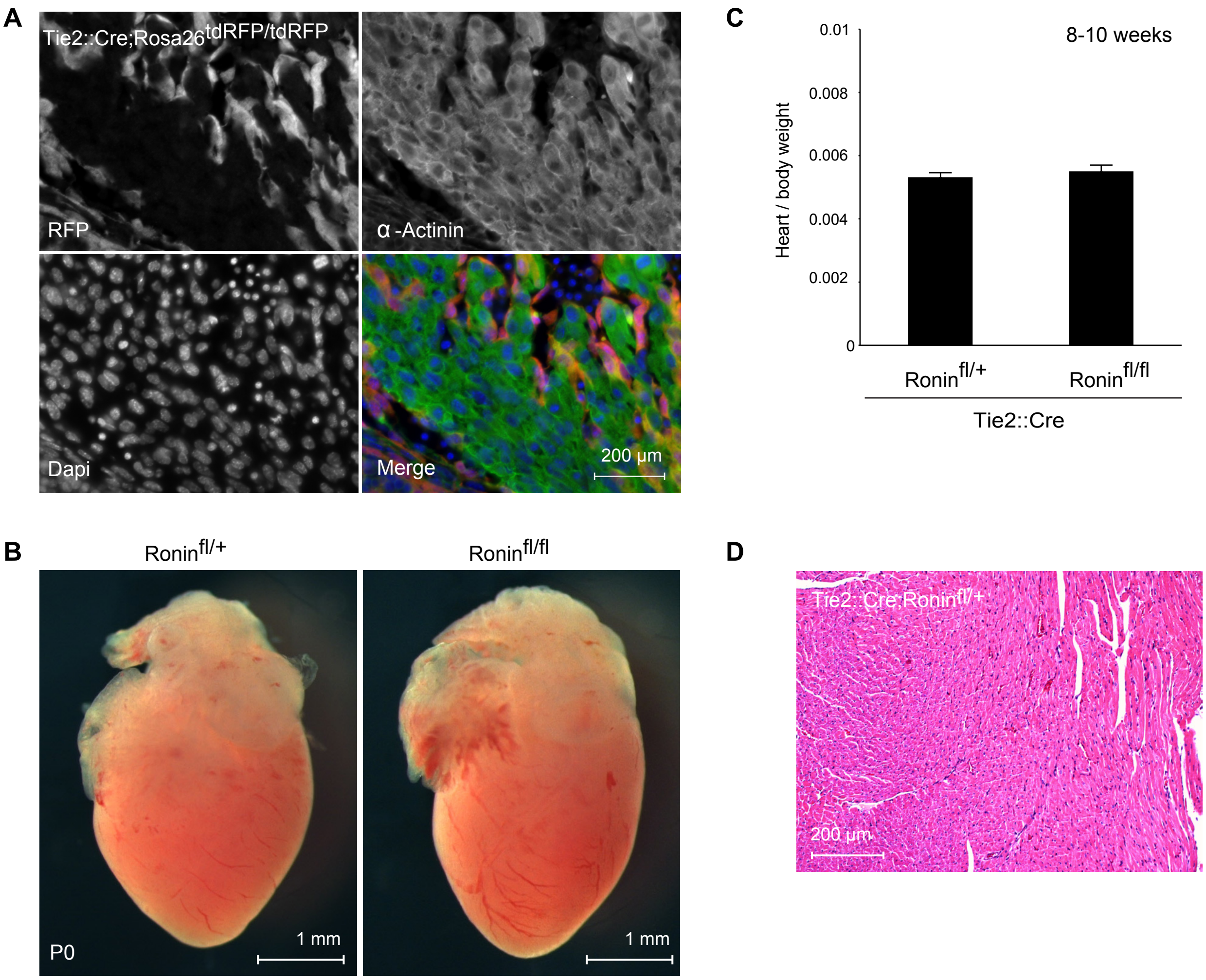

Figure S4. Characterization of *Tie2*::Cre driven *Ronin* knockout mice. Related to Figure 2. (**A**) *Tie2*::Cre animals were crossed with *Rosa26*$^{tdRFP/tdRFP}$ reporter animals. Immunofluorescence analysis of RFP-positive cells in heart tissue sections at E13.5 shows RFP signal exclusively in the endocardium, and not in the adjacent (α-Actinin positive) muscle. (**B**) At P0 (top) there are no overt macroscopic differences between hearts isolated from *Tie2*-driven *Ronin* knockout and those of heterozygous control animals. (**C**) The heart-to-body weight ratio in knockout and heterozygous controls shows no significant differences. (**D**) H&E staining of tissue sections obtained from heterozygous animals at 8 weeks of age shows well-ordered alignment of normal cardiomyocytes.

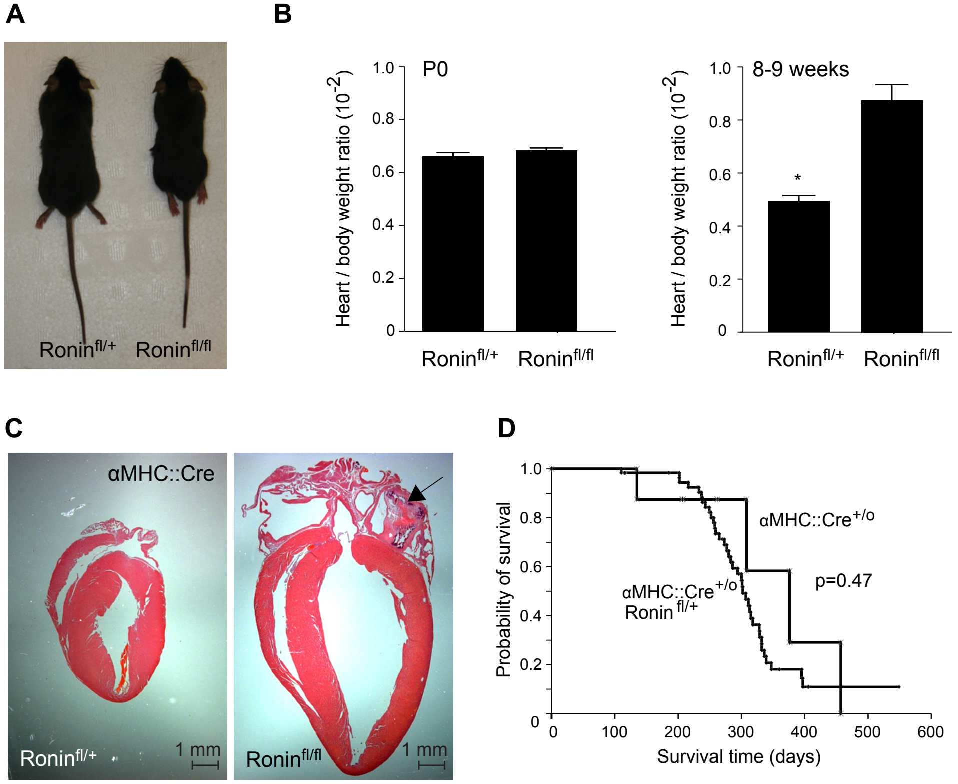

Figure S5. Phenotypic analysis of hearts isolated from *αMHC*-driven *Ronin* knockout animals at 12 weeks of age. Related to Figure 3. (**A**) Male knockout animals are significantly smaller than their heterozygous counterparts (26.6 g vs. 28.1 g; p=0.029 by t-test), possibly due to cardiac cachexia. (**B**) The heart-to-body weight ratio was identical in *Ronin* knockout animals and controls at P0 (left), but significantly higher in *Ronin* knockout animals by the age of 8-9 weeks (right). *p<0.05 by t-test. (**C**) Macroscopic histology of the heart of knockout animals at 12 weeks of age shows that all four chambers are dilated. Additionally, a very large thrombus (arrow) is apparent in the left atrium in comparison to heterozygous controls. (**D**) Kaplan-Meier analysis shows that the survival times of *αMHC*-driven heterozygous *Ronin* knockout animals do not differ significantly from *αMHC::Cre*-expressing animals. p-value was determined by log-rank test.

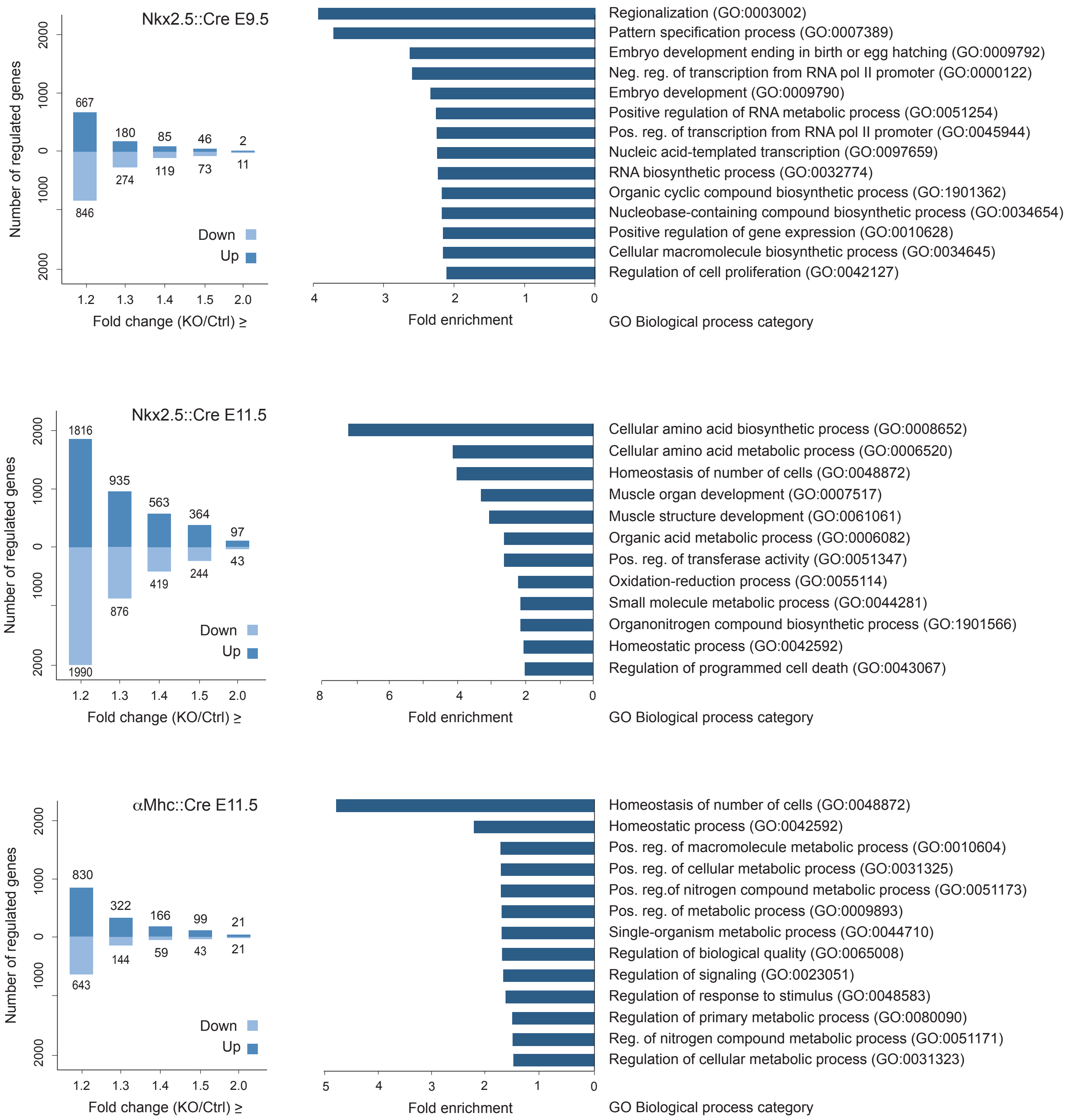

Figure S6. Analysis of differentially expressed genes in *Nkx2.5*- or *αMHC*-driven Ronin knockout compared with control heart tissue during embryonic development at E9.5 or E11.5. Related to Figure 5. Shown are the number of up- or downregulated genes (left) and selected phenotypically relevant gene ontology categories of biological processes (right), that are significantly enriched (with a p-value lower or equal to 0.5) using gene sets with a cutoff where at least 300 genes are present (1.3-, 1.5- or 1.3-fold, respectively, from top to bottom). Ctrl, control; KO, knockout; GO, gene ontology.

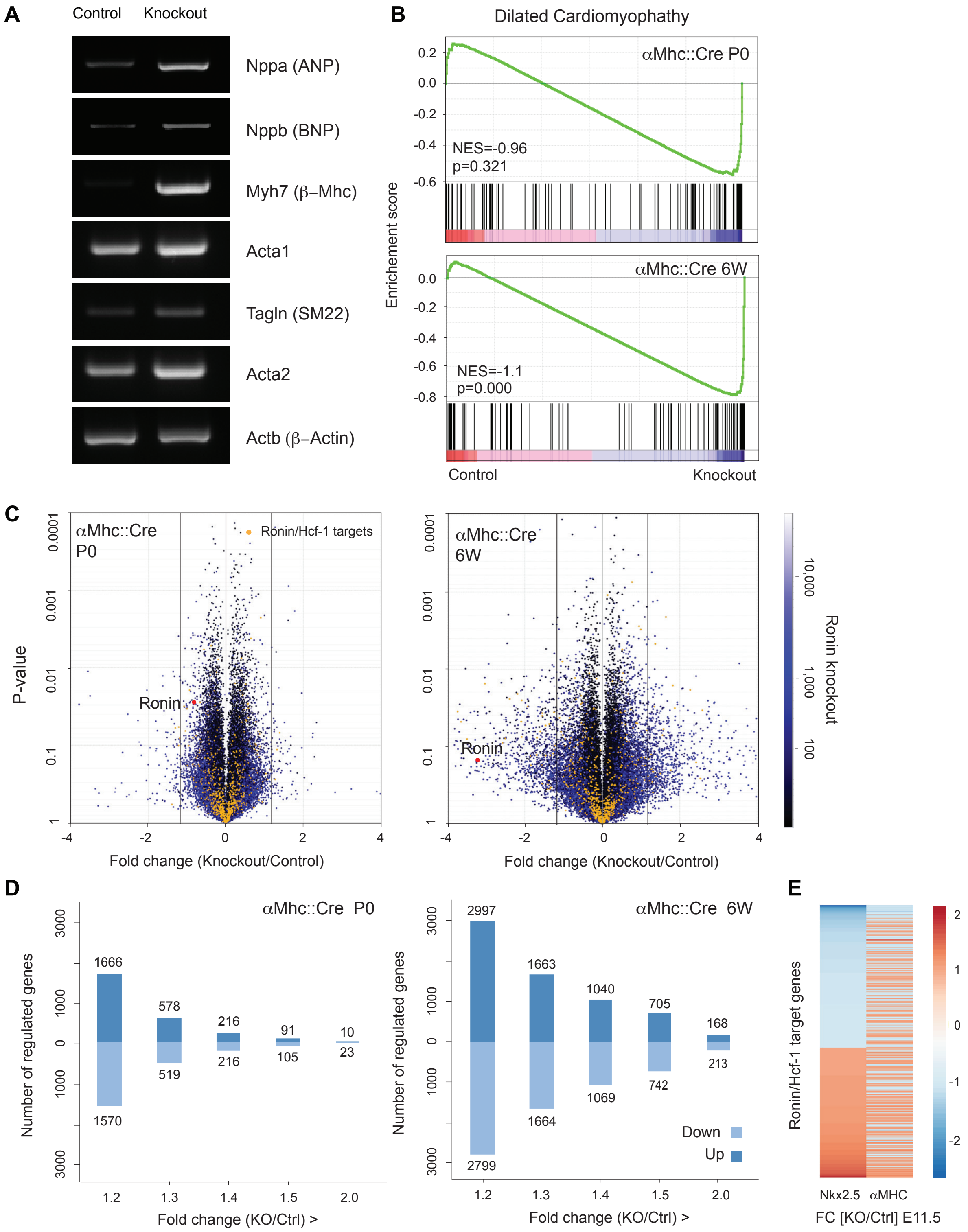

Figure S7. Gene expression analysis of hearts isolated from *Ronin* knockout animals. Related to Figure 5. (**A**) RT-PCR shows that selected markers of dilated cardiomyopathy are upregulated in heart tissue of *Ronin* knockout animals at 6 weeks of age. (**B**) GSEA analysis reveals the dysregulation of a gene set related to dilative cardiomyopathy at P0 after α*MHC*-driven *Ronin* knockout that becomes significantly enriched at 6 weeks of age. (**C**) Volcano plots of gene expression data obtained from microarray analyses at indicated time points. Yellow circles, Ronin/Hcf-1 targets. (**D**) Number of up- or downregulated genes in heart tissue after *Ronin* knockout when compared with controls. (**E**) Heatmap of Ronin/Hcf-1 target gene expression in heart tissue after *Ronin* knockout at E11.5. Target genes are sorted by fold change in *Nkx2.5*-knockout. FC, Fold change; NES, normalized enrichment score. Ctrl, control; KO, knockout; 6W, 6 weeks.

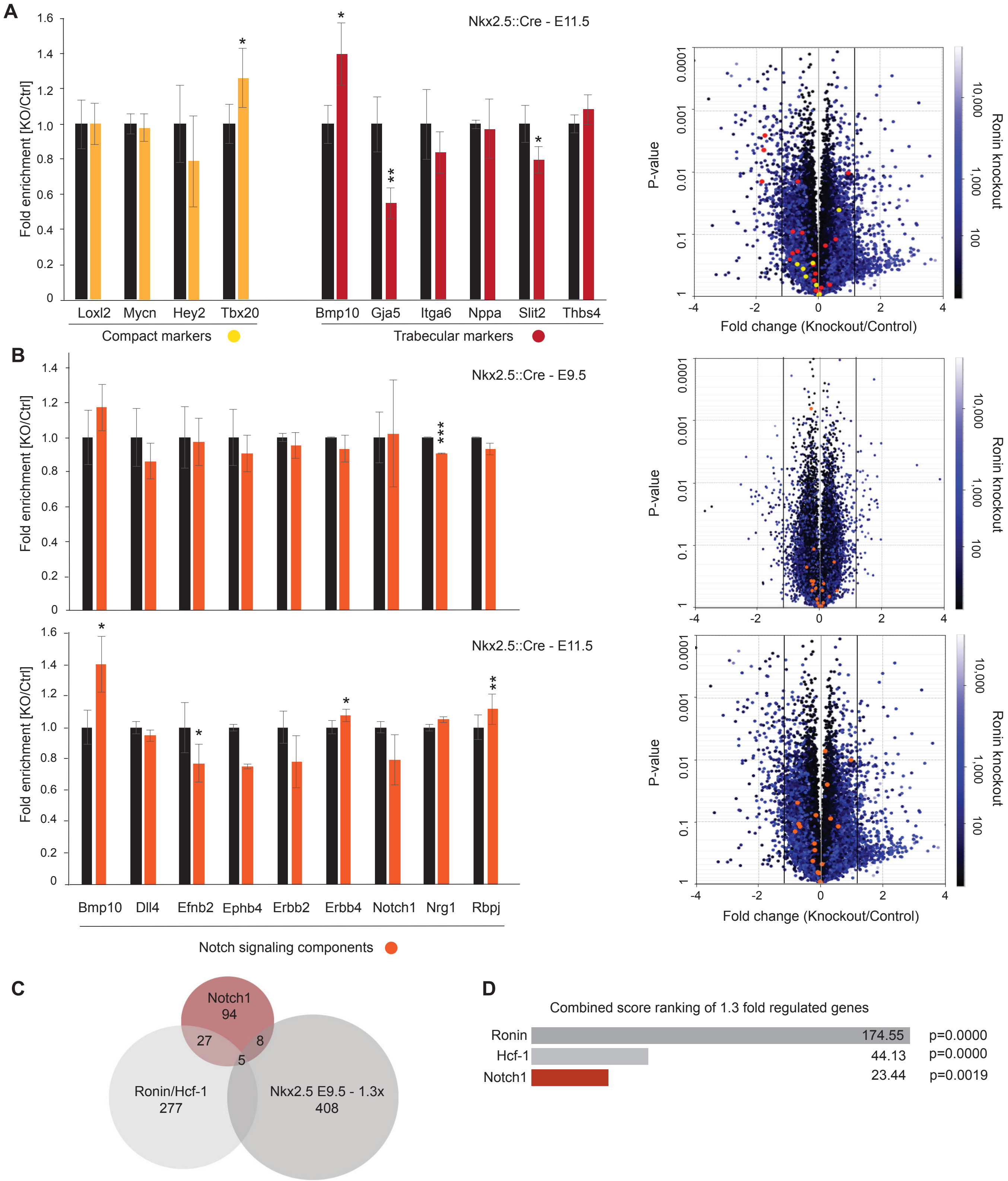

Figure S8. Differential gene expression analysis of selected gene sets at E9.5 and E11.5 of *Nkx2.5*-driven *Ronin* knock-out heart tissue. Related to Figure 5. (**A**) Markers specific for the myocardial compact or trabecular layers show mild changes in both layers at E11.5 after *Ronin* knockout (left). The gene probes are highlighted within the volcano plot (left) relative to all other gene probes detected in microarrays at E11.5. (**B**) Analyses of Notch signaling components do not reveal a specific direct involvement in the phenotypic changes observed after *Ronin* knockout at E9.5 (top) or E11.5 (bottom). Gene expression is illustrated as described in (A). (**C**) Venn diagram showing the overlap of Ronin/Hcf-1 with Notch1 targets (Chip enrichment analysis (ChEA) 2016 database set *NOTCH1_17114293_ChIP-ChIP_T-ALL _Human*) and the genes that are more than 1.3-fold up- or downregulated at E9.5. after *Nkx2.5*-driven *Ronin* knockout. (**D**) ChEA of genes that are up- or downregulated more than 1.3-fold in heart tissue at E9.5 after *Nkx2.5*-knockout. Shown are the combined score and p-value for each category. Ronin, ChEA 2016 database set: Thap11_20581084_ChIP-Seq_MESCs_Mouse; ChEA 2016 database set Hcf-1, HCFC1_20581084_-ChIP-Seq_MESCs_Mouse; Notch1, ChEA 2016 database set as in (C). *p<0.05; **p<0.01; ***p<0.001.

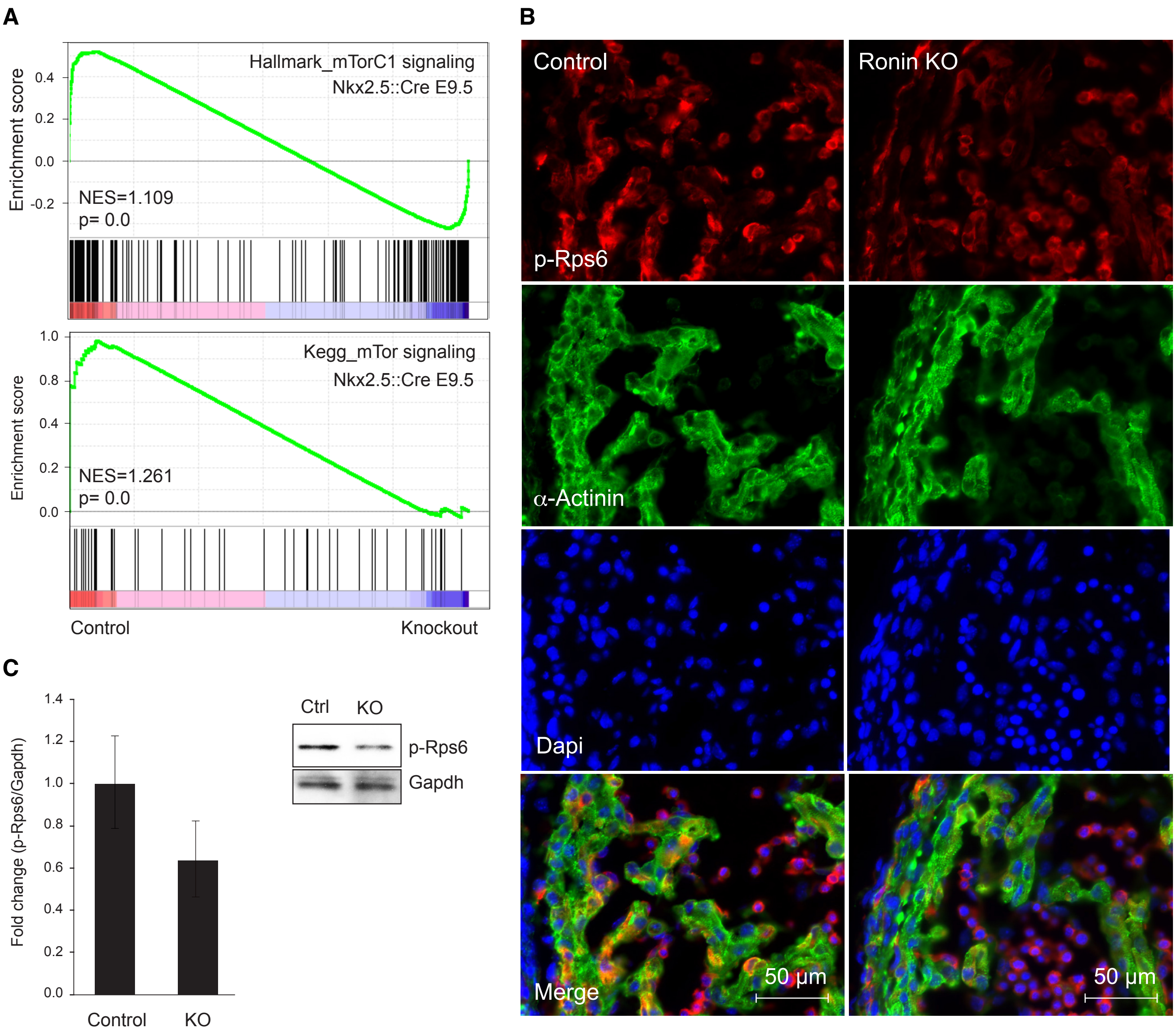

Figure S9. mTor activity is reduced in *Nkx2.5*-driven *Ronin* knockout animals at E11.5. Related to Figure 5. (**A**) Gene set enrichment analysis (GSEA) shows significant enrichment of the "Hallmark" and "Kegg" mTor signaling components in control tissue after *Nkx2.5::Cre*-driven knockout of *Ronin*. (**B**) Detection of the phosphorylation levels of the mTor target Rps6 at E11.5 by immunofluorescence reveals that heart tissue of *Nkx2.5*-knockout animals has lower Rps6 phosphorylation levels than heterozygous controls while α-Actinin or Gapdh remain at similar protein levels. Red, p-Rps6; green, α–Actinin; blue, Dapi. (**C**) Quantification of Western blot analysis confirms decreased phosphorylation of p-Rps6 upon *Ronin* knockout (n=3 ± SEM).

SUPPLEMENTAL EXPERIMENTAL PROCEDURES

Genotyping

The genotypes of all offspring were analyzed with DNA isolated from either the yolk sac of embryos or adult tail biopsies. The 5` and 3` primers amplifying *Nkx2.5::Cre*, *αMHC::Cre, Rosa25-tdRFP*, *Tie2::Cre* and the *Ronin*^{fl} alleles were: 5'-GATTA GCTTA AGCGG AGCTG GGTGT CC-3', 5'-GTTCT GGAAC CAGAT CTTGA CCTGC GTGGA-3'; 5'-GCCGC ATAAC CAGTG AAACA GCATT GC-3', 5'-ATGAC AGACA GAT CC CTC CT ATCTC C-3'; 5'-CTCAT CACTC GTTGC ATCAT CGAC-3', 5'-AAGAC CGCGA AGAGT TTGTC C-3'; 5'-T AAGC CTGCC CAGAA GACTC C-3', 5'-AAGGG AGCTG CAGTG GAGTA -3'; 5`-GCGGT CTGGC AGTAA AAACT ATC-3', 5'-GTGAA ACAGC ATTGC TGTCA CTT-3'; 5'-TACCC AGAGC GCTTG CGCTC ACC AG-3', 5'-TCCA G ATGAA GCTCG TCCTA AGCGA-3', respectively.

Hematoxylin-and-Eosin Staining

Adult heart and embryonic tissues were fixed in 10% (v/v) formalin and transferred to 70% (v/v) ethanol. The tissue was embedded in paraffin and 10-µm sections were stained with hematoxylin and eosin by the Histology Service of the Department of Pathology at Baylor College of Medicine.

X-gal Staining

Embryos and heart tissues were fixed in fixation buffer (0.2% [w/v] glutaraldehyde, 2 mM $MgCl_2$, and 5 mM EGTA) for 15 min, followed by three 15 min washes in detergent-containing buffer (2 mM $MgCl_2$, 0.01% [w/v] sodium deoxycholate, and 0.02% [v/v] NP-40). The tissue was then stained with X-gal solution (40 mg/ml X-gal in N,N, dimethylformamide mixed 1:40 with X-gal reaction buffer (100 µl 1M $MgCl_2$, 49.4 ml PBS, 82 mg potassium ferricyanide and 106 mg potassium ferrocyanide) for 4 – 5 hrs. They were washed three times in PBS, fixed in 4 % (v/v) paraformaldehyde overnight, and preserved in 70 % EtOH.

Electron Microscopy

Hearts were excised, fixed in glutaraldehyde, sectioned, and examined at the Department of Pathology at the Texas Children's Hospital.

Electrocardiography and Echocardiography

Electrocardiography (ECG) and echocardiography recordings were performed in the Mouse Phenotyping Core Facility at Baylor College of Medicine. ECG recordings were made with ECGenie, which provides a lead II ECG from the paws in conscious mice. Echocardiography was performed with a Visualsonic Vevo 770 Imaging System. Mice were anesthetized with isoflurane to maintain spontaneous breathing. The left ventricular ejection fraction and left ventricular internal end-diastolic diameter were measured.

ChIP-sequencing and ChIP-qPCR

Adapter ligation and ChIP-sequencing was performed as described previously (Dejosez et al., 2010). The ChIP-derived reads were aligned to the mouse genome (NCBI build 37, UCSC build mm9) using the FindPeaks software (UBC). Chip sequencing data were validated by ChIP-qPCR using Sybr Green in real time PCR analysis. The primers amplifying the *Apba3*, *Ctf-1*, *Mrpl34* and *Smarcal1* promoters were: 5'-TGAGT TTCAA GGGTC AGAGC -3', 5'-AACTC AGGCT TTAGG TCGTG -3'; 5'-AAAGC TGAAA GACTG GAGGG -3', 5'-TCCGA AGCCT GTATT TTGAG C-3'; 5'-GTGAG TGTTC AGAGA TACCC TG-3', 5'-TGTAT GACTT TCTGA GCCGC -3'; 5'-GCTAG GTAAG AAAGT GGGCT G-3', 5'-TCCTC TTGCC GTATT TGGTC -3', respectively. All samples were tested in triplicate and data were normalized to PCR signals of the corresponding input samples.

Bioinformatics Analyses

The logo reflecting the Ronin binding motif was created with Weblogo software (http://weblogo.berkeley .edu/). The Venn diagram was generated with the Venn Charts Google Developers online tool (https:// developers.google.com). GSEA was conducted with GenePattern (Broad Institute; Reich et al., 2006). The PANTHER tool (Thomas et al., 2003) was implemented to determine functional categories of the Ronin/Hcf-1 target genes or the genes that were differentially expressed after *Ronin* knockout. The chromatin enrichment analysis (ChEA) was performed with the web-based Enrichr tool (Kuleshov et al., 2016). The heat map in Figure S7 was produced with the ClustVis webtool (targets were sorted by fold change. The $H3K4me_3$ status between different gene sets was compared with the Genboree signal comparison tool (Bioinformatics Research Laboratory, Baylor College of Medicine; www.genboree.org). We used a linear regression of the $H3K4me_3$ signals of control and *Nkx2.5*-driven Ronin knockout signals over all gene promoters (Table S6). The results are annotated by z-scores. Positive z-scores indicate promoters with lower $H3K4me_3$ levels, negative z-scores indicate promoters with higher $H3K4me_3$ levels in *Ronin* knockout tissue by comparison with controls. Z-scores above 2 were considered significant.

Western Blot Analysis

Western blot analysis was performed as previously described (Dejosez et al., 2008). Primary antibodies were anti-phospho-S6 (2211, Cell Signaling; 1:1000) and anti-GAPDH (ab9485, Abcam; 1:2500).

Tunel Staining

Tunel staining was performed with the DeadEnd™ Colorimetric TUNEL System (Promega) in accordance to the manufacturers protocol.

Statistics

All values are presented as means ± SEM or SD as indicated. Statistical significance was evaluated with the unpaired Student's t-test for comparison of two means. The Kaplan-Meier method was used to estimate survival rates, while mortality was computed with the χ^2 test. A p-value of < 0.05 was considered to indicate significance.

SUPPLEMENTAL REFERENCES

Kuleshov, M.V., Jones, M.R., Rouillard, A.D., Fernandez, N.F., Duan, Q., Wang, Z., Koplev, S., Jenkins, S.L., Jagodnik, K.M., Lachmann, A., *et al.* (2016). Enrichr: a comprehensive gene set enrichment analysis web server 2016 update. Nucleic Acids Res *44* :W90-97.

Reich M., Liefeld, T., Gould, J., Lerner, J., Tamayo, P., Mesirov, J.P. (2006). GenePattern 2.0. Nat Genet *38*, 500-501.

Thomas, P.D., Kejariwal, A., Campbell, M.J., Mi, H., Diemer, K., Guo, N., Ladunga, I., Ulitsky-Lazareva, B., Muruganujan, A., Rabkin, S.*, et al.* (2003). PANTHER: a browsable database of gene products organized by biological function, using curated protein family and subfamily classification. Nucleic Acids Res *31*, 334-341.
