# Supplementary material for: Ronin Governs Early Heart Development by Controlling Core Gene Expression Programs
Source: Cell Rep. 2017 Nov 7;21(6):1562–73. doi: 10.1016/j.celrep.2017.10.036 (PMC5695914; doi:10.1016/j.celrep.2017.10.036)
Supplement: Document S2. Article plus Supplemental Information [file mmc5.pdf]

Cell Reports

Article

Ronin Governs Early Heart Development by Controlling Core Gene Expression Programs

Graphical Abstract

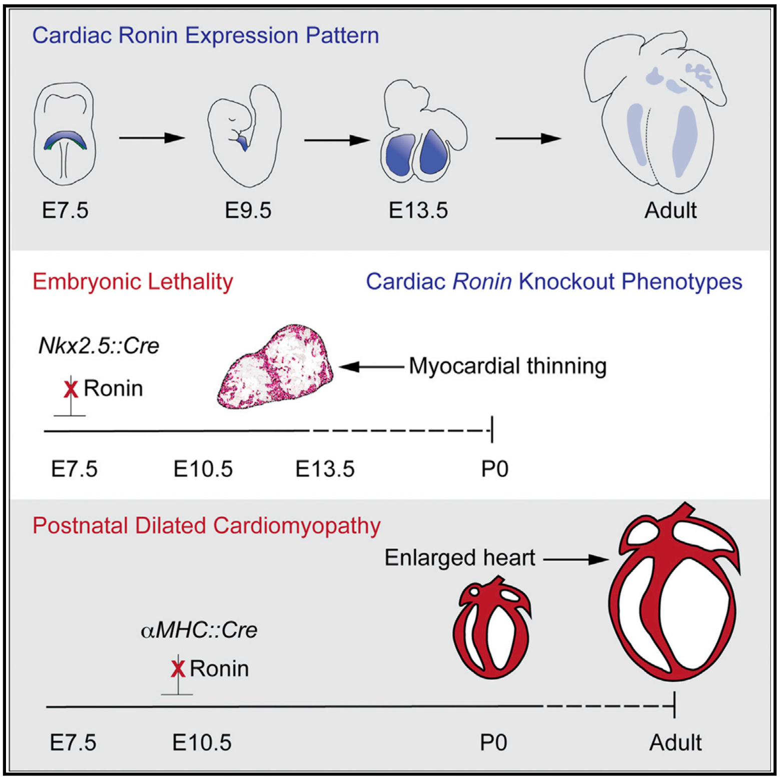

Highlights

- Ronin displays complex expression patterns during embryogenesis
- Ronin is critical for heart growth
- Ronin regulates genetic growth programs
- Ronin binding influences $H3K4me_3$ levels at target genes

Authors

Jun Fujita, Pablo Freire, Cristian Coarfa, ..., Michael D. Schneider, Marion Dejosez, Thomas P. Zwaka

Correspondence

marion.dejosez@mssm.edu (M.D.), thomas.zwaka@mssm.edu (T.P.Z.)

In Brief

Fujita et al. find that the transcriptional regulator Ronin (Thap11) appears to control embryonic heart development. Early heart-specific knockout of Ronin leads to growth defects in the developing heart and embryonic lethality, whereas slightly later Ronin loss results in severe dilated cardiomyopathy in the adult.

Data and Software Availability

GSE103959

CrossMark

Fujita et al., 2017, Cell Reports *21*, 1562–1573
November 7, 2017 © 2017 The Author(s).
https://doi.org/10.1016/j.celrep.2017.10.036

CellPress

OPEN ACCESS
CellPress

Cell Reports
Article

Ronin Governs Early Heart Development by Controlling Core Gene Expression Programs

Jun Fujita,[1] Pablo Freire,[2] Cristian Coarfa,[3] Ashley L. Benham,[4] Preethi Gunaratne,[5] Michael D. Schneider,[6] Marion Dejosez,[7,*] and Thomas P. Zwaka[7,8,*]

[1]Department of Cardiology, Keio University School of Medicine, Tokyo 160-8582, Japan
[2]Department of Cellular and Molecular Biology, Baylor College of Medicine, Houston, TX 77030, USA
[3]Molecular and Cellular Biology Department, Baylor College of Medicine, Houston, TX 77030, USA
[4]Stem Cell Engineering Department, Texas Heart Institute at St. Luke's Episcopal Hospital, Houston, TX 77225, USA
[5]Department of Biology and Biochemistry, University of Houston, Houston, TX 77204, USA
[6]British Heart Foundation Centre of Research Excellence, National Heart and Lung Institute, Imperial College London, London SW7 2AZ, UK
[7]Black Family Stem Cell Institute and Department of Cell, Developmental and Regenerative Biology, Icahn School of Medicine at Mount Sinai, New York, NY 10029, USA
[8]Lead Contact
*Correspondence: marion.dejosez@mssm.edu (M.D.), thomas.zwaka@mssm.edu (T.P.Z.)
https://doi.org/10.1016/j.celrep.2017.10.036

SUMMARY

Ronin (THAP11), a DNA-binding protein that evolved from a primordial DNA transposon by molecular domestication, recognizes a hyperconserved promoter sequence to control developmentally and metabolically essential genes in pluripotent stem cells. However, it remains unclear whether Ronin or related THAP proteins perform similar functions in development. Here, we present evidence that Ronin functions within the nascent heart as it arises from the mesoderm and forms a four-chambered organ. We show that Ronin is vital for cardiogenesis during midgestation by controlling a set of critical genes. The activity of Ronin coincided with the recruitment of its cofactor, Hcf-1, and the elevation of H3K4me$_3$ levels at specific target genes, suggesting the involvement of an epigenetic mechanism. On the strength of these findings, we propose that Ronin activity during cardiogenesis offers a template to understand how important gene programs are sustained across different cell types within a developing organ such as the heart.

INTRODUCTION

Even early in development, the heart siphons primordial blood while continuing its journey through the difficult rearrangements and growth needed for it to become a fully configured organ (Rosenthal and Richard 2010). During this complex process, missteps occur frequently (1%–2% of newborns have congenital heart defects; Hoffman and Kaplan, 2002) and the adult heart shows little regeneration (Rosenthal and Richard 2010). Molecularly, cardiogenesis entails coordinated efforts among diverse transcription factors (Olson, 2006). Some of these factors, such as Nkx2.5, assign cell identity early and broadly (Lyons et al., 1995); others, such as Tbx5, act later and in more bound cellular neighborhoods within the cardiac anlagen (Hiroi et al., 2001), whereas still others, like GATA4, GATA6 (Kuo et al., 1997; Molkentin et al., 2000), and various epigenetic enzymes (e.g., p300, histone deacetylases [HDACs], and Brg1/Brm-associated factor [BAF]) (Chang et al., 2004; Lickert et al., 2004; Shikama et al., 2003), coregulate heart development. Although acting with precision, Nkx2.5, Tbx5, and related factors are short-lived and space restricted (Wamstad et al., 2012), so it is challenging to consider a mechanism that might account for very broad transcriptional regulation within an organ composed of diverse cell types. A baton-like handoff of signals would solve this issue in part, but the nascent cardiac tissue may also require other factors that safeguard the robustness of critical mRNAs that cross developmental boundaries, thereby ensuring the coordination and continuity of transcription.

Ronin (Thap11) is an attractive candidate for such a broad regulatory role in cardiogenesis. Discovered in embryonic stem cells and named after a masterless samurai because of its lack of any apparent relationship to the known "master" regulators of pluripotency, it is essential for this state, both in vitro and in vivo (Dejosez et al., 2008). Critically, Ronin binds to a hyperconserved promoter sequence (5′-CTGGGARWTGT-3′) present in many genes and spanning various cellular rubrics (Dejosez et al., 2010). It controls these genes in a yet-undetermined manner in a variety of settings, including the developing eye (Poché et al., 2016), neural crest and craniofacial development (Achilleos et al., 2017a), a cobalamin deficiency syndrome impacting the brain (Achilleos et al., 2017b), hematopoiesis (Kong et al., 2014), cancer cells (Parker et al., 2012), and during the transition from the primed to naive pluripotent stem cell state (Durruthy-Durruthy et al., 2016). Here, we identify a broad-ranging and dynamic binding pattern that allows Ronin to control gene expression across diverse cell types and temporal hallmarks in the developing heart.

RESULTS

Ronin Displays a Complex and Dynamic Expression Pattern during Embryogenesis

For a transcriptional regulator to exert broad time- and space-specific control of gene expression across cell types, its expression

 © 2017 The Author(s).
This is an open access article under the CC BY license (http://creativecommons.org/licenses/by/4.0/).

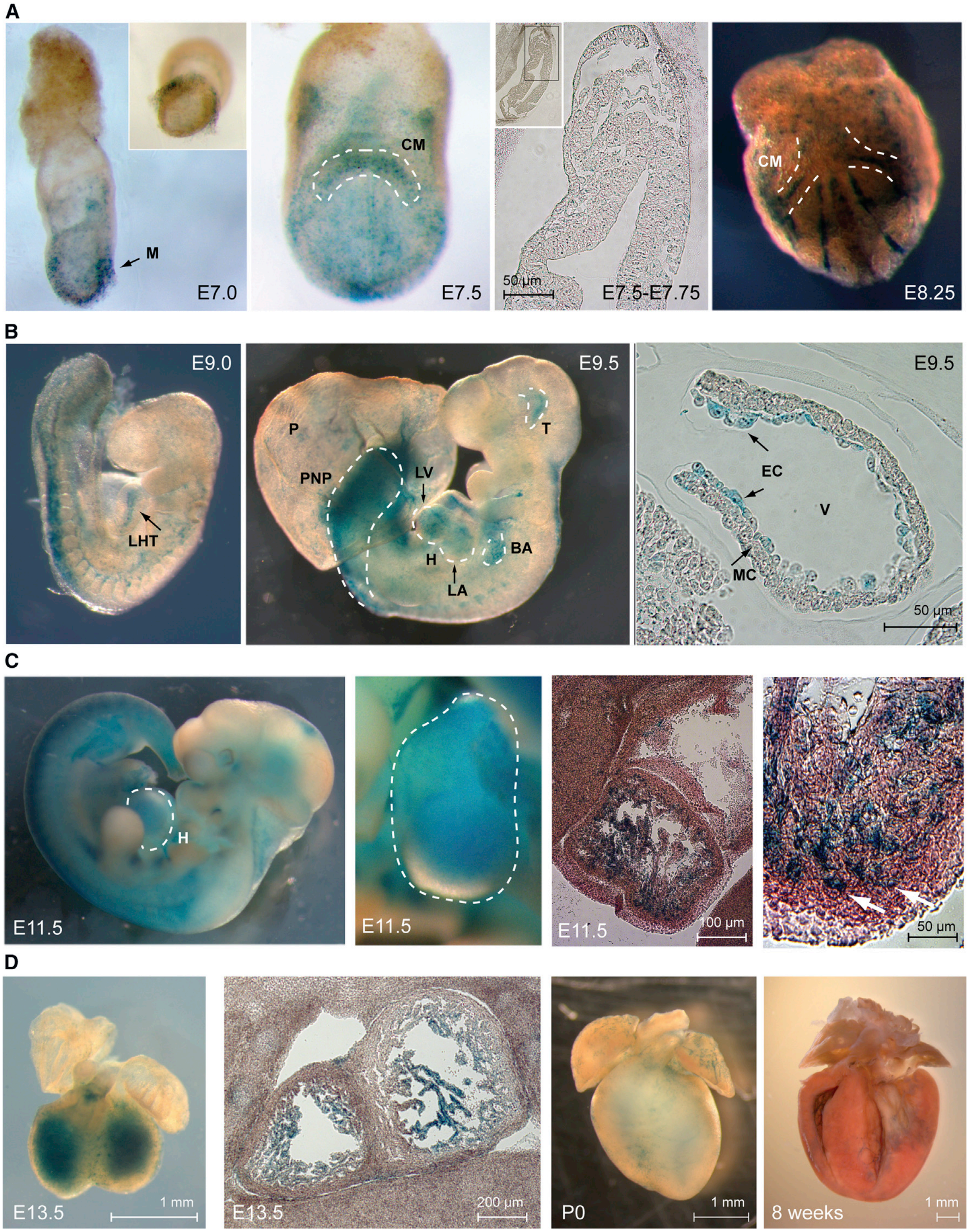

Figure 1. Ronin Is Expressed Early in Cardiogenesis Based on lacZ Expression at the Indicated Developmental Stages

(A) At E7.0, Ronin is expressed in the embryonic mesoderm and then appears in the cardiac crescent (E7.5–8.25).

(B) It is found in the looped heart tube at E9.0 (left) and the inner layers of the nascent heart structure, the thalamus, branchial arches, and posterior neuropore at E9.5 (middle). Microscopically, hearts at E9.5 (right) show their highest Ronin expression levels in the endocardium and far less in the myocardium.

(C) At E11.5, Ronin expression peaks overall (left) and large portions of the heart appear positive (second panel from left). Histologically, the myocardial trabeculae stain positive for Ronin, whereas no signal is seen within the compact layer (right two panels). Note the inward-reaching myocytes (white arrows).

(legend continued on next page)

must be conducive to this function. We therefore tracked Ronin expression during development using *Ronin::lacZ* reporter mice (Figures 1 and S1). Ronin expression underwent a sequence of complex yet spatially well-defined steps as it re-emerged in the mesoderm (E7.0) after an implantational hiatus (Dejosez et al., 2008), only to be restricted to the cardiac crescent shortly thereafter (E7.5–E8.25) and then to the looped heart tube (E9.0), always bypassing cell-type boundaries. By E9.5, Ronin dominated the endocardial layer of the heart and was strongly expressed in other embryonic structures, such as the thalamus, branchial arches, and posterior neuropore. Ronin expression peaked at E11.5, when lacZ staining was visible throughout larger portions of the heart, most of the nervous system, and many other organs. Histological examination of the heart at this stage showed that Ronin expression was most prominent in the myocardial trabecular layer. This pattern remained essentially unchanged at E13.5, but at the end of trabeculation, Ronin levels had declined and remained detectable in only a small number of cells in the subendocardial layer at P0 (postnatal day 0). String-like subendocardial networks of Ronin-positive cells appeared to persist well into adulthood (Figures 1D and S1A), whereas histological sections of adult *Ronin::lacZ* hearts betrayed little-to-no Ronin expression in cardiac myocytes (Figure S1B). These observations suggest that Ronin may direct critical genetic programs not only in the preimplantation embryo (Dejosez et al., 2008), but throughout development and, to a lesser extent, in the adult as well.

Ronin Is Critical for Heart Growth

To gauge the involvement of Ronin in heart development in greater detail, we crossed mice homozygous for the floxed Ronin allele with *Nkx2.5::Cre* animals (Moses et al., 2001) to generate a cardiac progenitor-specific knockout model. The functionality of *Nkx2.5::Cre*-mediated gene ablation was independently confirmed by breeding *Nkx2.5::Cre* mice with *Rosa-26-loxP-STOP* reporter animals (Luche et al., 2007), where we observed red fluorescent protein (RFP)-positive cells in the offspring as early as E7.5 in the cardiac crescent (Figure S2A). Additionally, qRT-PCR analysis confirmed significant reduction of Ronin expression after knockout (Figure S2B). *Nkx2.5::Cre; Ronin*$^{fl/+}$ control embryos lacked any discernible phenotype, whereas most *Nkx2.5::Cre; Ronin*$^{fl/fl}$ embryos died between E11.5 and E13.5 (Figures 2A and S2C). The E13.5-knockout heart exhibited a proper configuration, including unimpaired endocardial and epicardial linings, but the ventricular walls appeared undernourished and were substantially less dense than those of *Nkx2.5::Cre; Ronin*$^{fl/+}$ control embryos (Figure 2A, right). This phenotype could reflect the failure of individual cells to grow, a proliferation defect, or perhaps increased apoptosis. To distinguish among these possibilities, we studied knockout hearts at E11.5, observing a decline in the number of proliferating cells, particularly in the trabecular layer of the heart, accompanied by a slight, not statistically significant decrease in apoptosis (Figure S3).

The most parsimonious explanation for our results is that *Ronin* knockout directly triggers a growth defect in cardiac myocytes, leading to altered proliferation and eventually cell death, rather than exerting a direct effect on developmental cell fate decisions. Alternatively, the defect might first emerge in the endocardium and then spread to the heart muscle. This possibility is supported by our previous observation that some endocardial progenitor subsets originate from Nkx2.5-positive cardiogenic mesoderm (Stanley et al., 2002). To test for such an initial endocardial defect, we removed *Ronin* from the earliest (E8.25) endocardial cells (Kisanuki et al., 2001) using a *Tie2::Cre*; *Ronin*$^{fl/fl}$ cross (Figures 2B and S4). Neither embryos nor adult animals resulting from this cross showed any phenotype, arguing against an endocardial origin for the lethality seen in *Nkx2.5::Cre; Ronin*$^{fl/fl}$ embryos. We also considered whether faulty epicardial signaling could be a contributing factor, but this scenario seems unlikely given that only a minute fraction of the epicardial cells expressed Ronin.

Because *Nkx2.5::Cre* removed *Ronin* from the developing heart at a very early time, we next sought to extend our observations to more mature cardiac myocytes using *αMHC (myosin heavy chain)::Cre* as a driver because αMHC is expressed slightly later than Nkx2.5 during embryonic development. Again, we first tested the penetrance of Cre-mediated deletion by crossing the *αMHC::Cre* mice (Agah et al., 1997) with *Rosa-26-loxP-STOP* reporter animals (Luche et al., 2007). Although no Cre activity was detected at E8.0, from E10.5 on, a large proportion of the heart was RFP positive, suggesting *αMHC::Cre*-mediated conditional deletion of *Ronin* as early as E10.5, an effect that is sustained thereafter, as seen in sections at E13.5 or at 5 weeks of age (Figure S2D). When we crossed *Ronin*$^{fl/fl}$ animals with *αMHC::Cre* transgenic mice, the loss of *Ronin* did not yield any discernible cardiac growth defect or other abnormality during gestation (Figure 3A). The pups resulting from this cross were born at normal Mendelian frequencies (Figure S2C) and had apparently normal hearts at birth. However, they became moribund at 3 months of age and none survived beyond 250 days (Figure 3B). The survival curves of control animals (*αMHC::Cre; Ronin*$^{fl/+}$) mirrored those of age-matched *αMHC::Cre* mice (Figure S5), ruling out Cre toxicity as a cause of the adult phenotype. The sick mice all had dilated cardiomyopathy (DCM) with characteristic echocardiography (ECG) changes, including bradycardia, prolonged QRS intervals, and long QT waves (Figures 3C–3E). Their left ventricles were dilated, pumped with a low ejection fraction (Figure 3F), and showed other signs of DCM on echocardiography (Figure S5). Postmortem examination revealed that *αMHC::Cre* mice had enlarged hearts at 12 weeks of age (Figure 3G). Histologically and ultrastructurally, the heart texture was disordered and featured interstitial fibrosis, nuclear enlargement, G-band streaming, and myocyte degeneration (Figure 3H). Additionally, we confirmed the elevated expression of known markers of heart failure by RT-PCR (Figure S7A).

(D) The Ronin expression pattern remains essentially unchanged at E13.5, but Ronin expression falls after completion of trabeculation, with only a small ring of Ronin-positive cells (near the subendocardial layers in both ventricles) remaining at P0 and at 8 weeks of age (note that the heart is cut along the midsection). BA, branchial arches; CM, cardiac mesoderm; EC, endocardium; H, heart; LA, left atrium; LHT, looped heart tube; LV, left ventricle; M, mesoderm; MC, myocardium; P, placenta; PNP, posterior neuropore; T, thalamus; V, ventricle. See also Figure S1.

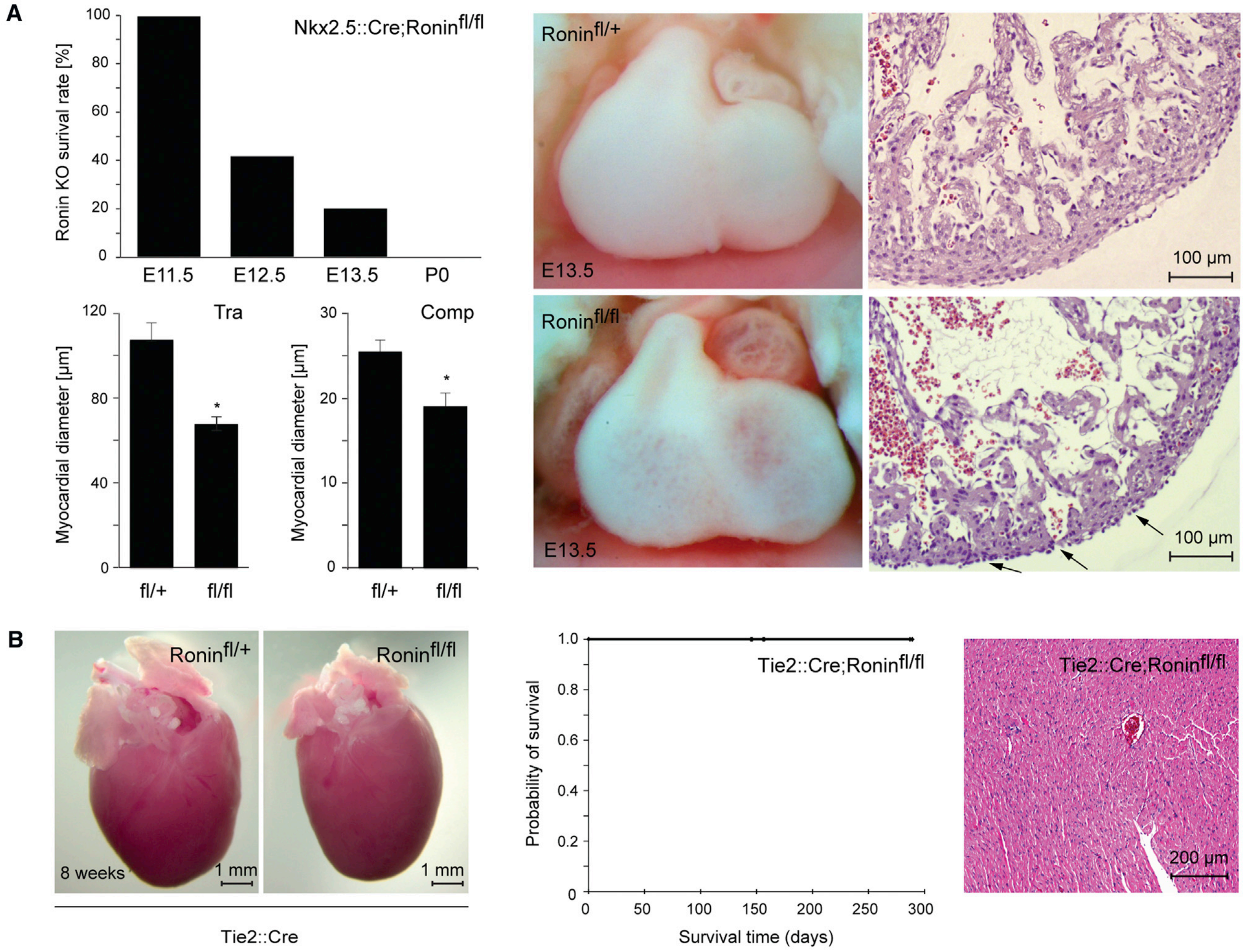

Figure 2. Early Ablation of *Ronin* Causes Cardiac Defects and Embryonic Lethality

(A) After *Nkx2.5*-driven *Ronin* ablation, more than half of the knockout embryos died by E12.5, three-fourths were dead by E13.5, and all embryos died before birth (left, top; n = 49, 59, or 79 at E11.5, E12.5, or E13.5, respectively; $p = 2.7 \times 10^{-6}$ by χ^2 test). Bright-field microscopy of the complete heart (middle) and histological analyses of H&E stained sections (right) revealed much thinner chamber walls in *Nkx2.5*-driven *Ronin* knockout hearts (bottom) than in heterozygous controls at E13.5 (top). Black arrows indicate ultra-thin areas of the myocardium. Comparison of the diameters of the trabecular (Tra) and compacted (Comp) myocardial layers with controls (left, bottom two panels) shows significantly decreased results in both layers of the *Nkx2.5*-driven *Ronin* knockout hearts (*$p < 0.05$ by t test). Values are reported as means ± SEM (n = 5 per group).

(B) At 8 weeks of age, there are no overt macroscopic differences between hearts isolated from *Tie2*-driven *Ronin* knockout and those of heterozygous control animals (left two panels). Kaplan-Meier survival analysis of *Tie2*-driven *Ronin* knockout animals. None of the animals died postnatally in the first 9 months of life (n = 13, middle panel). H&E staining of tissue sections obtained from knockout animals at 8 weeks of age shows well-ordered alignment of normal cardiomyocytes (right panel).

See also Figures S2–S4.

Thus, *Ronin* knockout by *Nkx2.5* and *αMHC* approaches produced diametrically opposed effects on the developing heart.

Ronin Regulates Heart Growth Programs

The broad though still dynamic expression pattern of Ronin offered a clue as to why the *Nkx2.5*- and *αMHC*-related phenotypes were so different because it suggested that Ronin may modulate a spectrum of vital genes across cell types for only a limited time. To test this hypothesis, we used chromatin immunoprecipitation sequencing (ChIP-seq) analyses of cardiac tissues extracted from E11.5 embryos to map Ronin's target gene repertoire (Table S1). We found that the binding of Ronin overlapped strikingly with that of its cofactor, Hcf-1, underscoring the close relationship between these two proteins (Figure 4A; Table S1). A DNA motif affiliated with Ronin binding (YGGGNNNYRTAGT; Figure 4B) was typically located proximal to the transcriptional start sites ($p = 1.0 \times 10^{-15}$; Figure 4C), identical to the Ronin binding signature found in pluripotent stem cells (Dejosez et al., 2010), retinal progenitors (Poché et al., 2016), and cancer cells (Parker et al., 2012). The genes targeted by Ronin/Hcf-1 largely belonged to metabolism-related categories, including primary metabolism, nucleic acid metabolism,

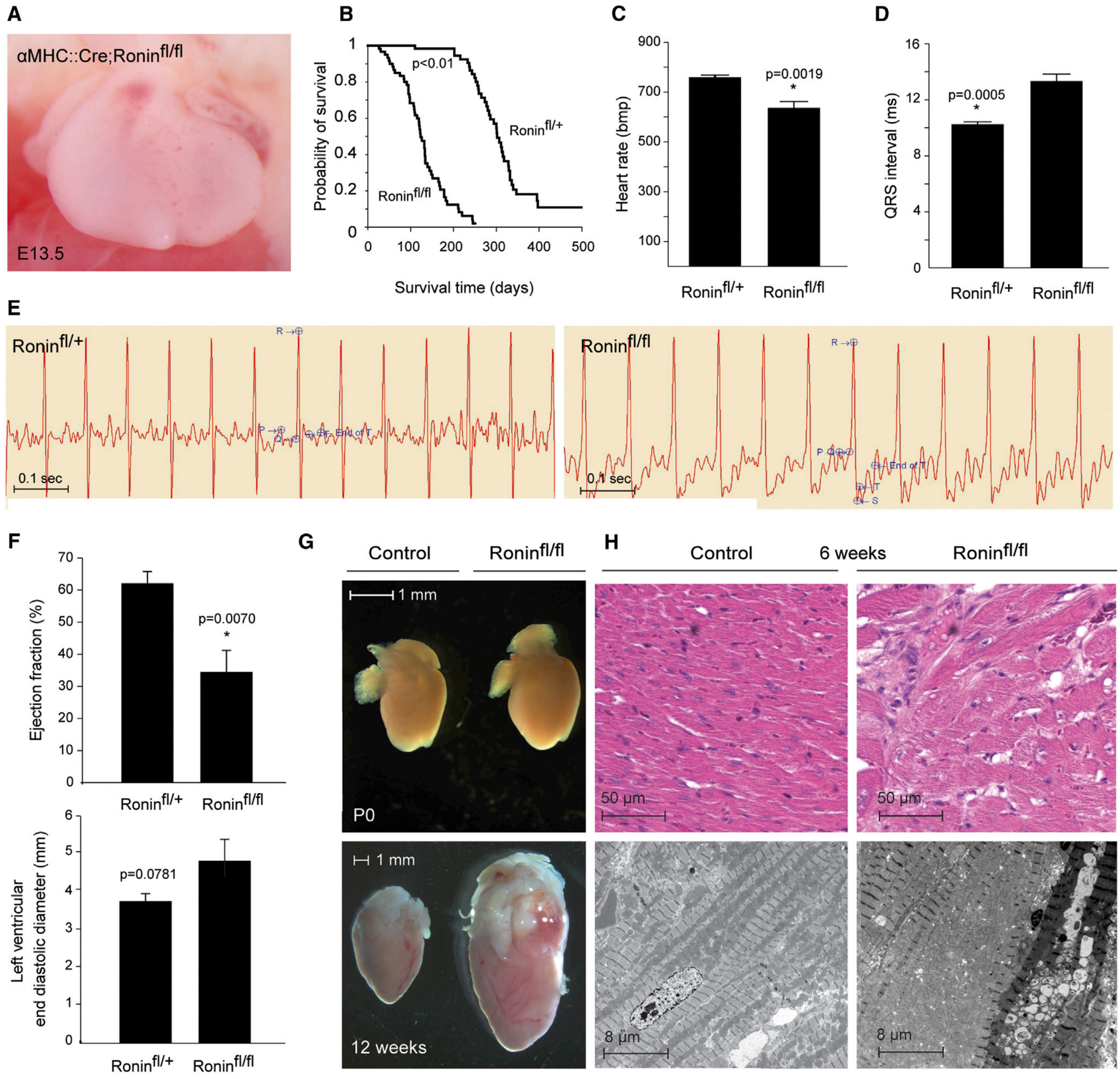

Figure 3. Midgestational Ablation of *Ronin* Leads to Postnatal Dilated Cardiomyopathy in Adult Mice

(A) Bright-field photomicrograph shows a normal-looking heart at E13.5 after *αMHC*-driven knockout of *Ronin*.

(B) Kaplan-Meier analysis shows that all mice with *αMHC*-driven *Ronin* knockout died before 9 months of age; the mean survival times for control (n = 60) and *Ronin* knockout (n = 62) mice was 262.8 days versus 123.6 days ($p = 9.9 \times 10^{-20}$ by log-rank test).

(C–E) Determination of heart rate (C) and QRS interval (D) following electrocardiography (E) demonstrate that animals with *αMHC*-driven *Ronin* knockout have bradycardia and a prolonged QRS interval. Bmp; beats per minute.

(F) Echocardiography reveals that animals with *Ronin* knockout have a significantly lower ejection fraction, whereas the ventricular end diastolic diameter appears elevated when compared with heterozygous controls. Data are reported as mean ± SEM (n = 5 per group).

(G) Neonatal heart size (P0) did not differ between *αMHC*-driven knockout and control animals (top), but at 12 weeks of age, the hearts of knockout animals were clearly enlarged (bottom).

(H) H&E staining (top) of heart tissue sections of *αMHC*-driven 6-week-old knockout mice showed hypertrophic cardiomyocytes and lymphocyte infiltration. A more detailed electron microscopic comparison of conditional knockouts with control animals (bottom) revealed fibrosis, increased numbers of mitochondria of various sizes, loss of granules, enlarged, hypertrophic, and disorganized nuclei, and thick Z-bands.

See also Figures S2 and S5.

and protein metabolism and transport (Figure 4D; Table S1). Together, our results suggest that the growth and metabolic pathways controlled by Ronin are indispensable for normal heart development during the E9.5–E11.5 interval and less essential thereafter.

To pursue this hypothesis further and learn if there is a temporal component to the Ronin-mediated regulation of target genes, we scrutinized global gene expression profiles in normal and knockout heart tissues at different time points. The initial gene expression changes were subtle; only around 100 genes were regulated more than 1.5-fold (*Nkx2.5* knockout at E9.5 or *αMHC* knockout at E11.5), with only a few genes exceeding the 2-fold threshold (Figures 5A–5C and S6; Table S2). These changes translated to more pronounced effects at later developmental stages (Figures 5 and S7). As shown by gene set enrichment analysis (GSEA), a gene signature specific to dilated cardiomyopathy emerged at P0 in *αMHC* knockout hearts and had become significant by 6 weeks (Figure S7B). Gene ontology (GO) analyses revealed that *Ronin* knockout-related gene expression changes were most prominent in metabolic categories at all stages, overlapping with processes involved in organ development (*Nkx2.5* driven) or heart function at later time points (*αMHC* driven; Figure S6; Table S2). The results of our analyses agree with previous findings in the developing retina (Poché et al., 2016), in which *Ronin* knockout led to impaired cell proliferation and increased cell death, most likely due to dysregulation of genes involved in metabolic processes and mitochondrial function in particular. Indeed, the mitochondrial gene signature that was discovered to respond to *Ronin* knockout in the retina was similarly downregulated after *Ronin* knockout in the heart. The affected genes were among the most strongly regulated genes in our experiments (Figures 5A–5C).

Comparison of our molecular findings with the corresponding phenotypes suggested that the *Ronin* knockout-related transcriptional changes during early heart development are highly detrimental, whereas those due to delayed knockout lead to more subtle changes that are tolerated for prolonged times. A clue to the mechanism of these disparate outcomes came from the observation that the expression levels of Ronin target genes fluctuate significantly between E9.5 and E11.5 (Figure 5D, bottom), but not earlier (between E7.5 to E9.5; Figure 5D, top). Moreover, the expression of Ronin target genes varied far more after *Nkx2.5*-triggered knockout than after *αMHC* ablation at E11.5 (Figures 5E, S7C, and S7D; Table S2). Sorting the Ronin/Hcf-1 target genes by expression level after *Nkx2.5* knockout indicated that genes dysregulated after *αMHC* knockout differ from those in *Nkx2.5*-knockout hearts (Figures 5F and S7E). Additional GSEA (Figure 5G; Table S2) confirmed these findings. Together, our results show that *Ronin* depletion causes the malfunction of vital developmentally timed genes with a strong bias toward early ontogenic episodes, which helps to explain the attenuated phenotype seen after *αMHC* knockout.

We also examined gene expression changes among markers specific for the myocardial trabecular or compact layer (Li et al., 2016; Luxán et al., 2013) in *Ronin* knockout hearts (Figure S8A), but this analysis did not indicate an effect on either layer in particular. It is noteworthy that expression of Bmp10, which is expressed in the trabecular myocardium to sustain proliferation, was slightly elevated, possibly reflecting a compensatory mechanism to counteract the decline in trabecular cardiomyocytes. In addition, the gene expression profile of *Ronin* knockout heart tissue did not indicate a direct disturbance of Notch signaling pathway components, which are intimately involved in the initial formation of the myocardial layers (Figure S8B) early after *Nkx2.5* knockout of *Ronin* at E9.5. Nonetheless, we found that Notch1 and Ronin/Hcf-1 target genes do overlap to some degree. Chip enrichment analysis (ChEA) showed that 32 of our Ronin/Hcf-1 targets are also part of the significantly enriched Notch1 target gene set. Furthermore, a total of 13 Notch1 target genes were regulated more than 1.3-fold in heart tissue at E9.5 after *Nkx2.5* knockout of *Ronin* (Figures S8C and S8D). This suggests that Ronin might act in parallel or downstream of Notch and presumably other canonical signaling pathways to control growth and proliferation.

Additionally, we investigated potential mechanistic links between the transcriptional changes induced by *Ronin* knockout and pathways of cell growth and metabolism in cardiac myocytes. Our PANTHER analyses (Figure 4D; Table S1) suggested that mTor (mammalian target of rapamycin), a master regulator of cell growth and metabolism (Holz et al., 2005; Ma and Blenis, 2009), could contribute to the altered growth phenotype observed in our study. Compelled by additional GSEA results showing dysregulated expression of mTor-related gene signatures as early as E9.5 after *Nkx2.5* knockout (Figure S9A), we examined the effects of *Ronin* ablation on mTor activity. At E11.5, control embryos stained positive for the prototypical target of mTor kinase activity, p-Rps6 (cytoplasmic phosphorylated ribosomal protein S6), but *Nkx2.5::Cre; Ronin*$^{fl/fl}$ embryos showed little p-Rps6 positivity in cardiomyocytes (Figure S9B). Decreased phosphorylation of Rps6 after *Ronin* knockout was confirmed by western blot analysis (Figure S9C), suggesting that Ronin affects mTor signaling in the heart. Hence, the growth defects seen upon early knockout could be a direct consequence of transcriptional influences on mTor pathway components or perhaps a secondary effect of altered amino acid metabolism (Dejosez et al., 2010).

Ronin Binding Correlates with H3K4me$_3$ Levels

If, in fact, Ronin controls cell growth and metabolism during heart development, how exactly does it achieve this regulatory end point? Although Ronin lacks a specific transactivation domain, it can act through its coactivator Hcf-1 (Dejosez et al., 2008, 2010), which mobilizes H3K4 methyltransferases with consequent deposition of H3K4me$_3$ histone marks (Tyagi et al., 2007). Such marks could persist at gene loci critical to cardiac growth, thus facilitating rapid cell proliferation during development. To test this hypothesis, we performed ChIP-seq studies on E11.5 control and *Nkx2.5*-driven *Ronin* knockout embryonic heart tissues using antibodies against H3K4me$_3$ (Table S1). Strikingly, GO analysis of all gene promoters with significantly lower methylation levels after *Ronin* knockout (Z score > 2) revealed that a number of the associated genes are involved in cardiac function (Figure 6A; Table S3). When we focused on Ronin/Hcf-1 target genes, we found that most of the downregulated Ronin target genes showed lower levels of H3K4me$_3$ compared to (1) genes that were downregulated but not bound by Ronin; or

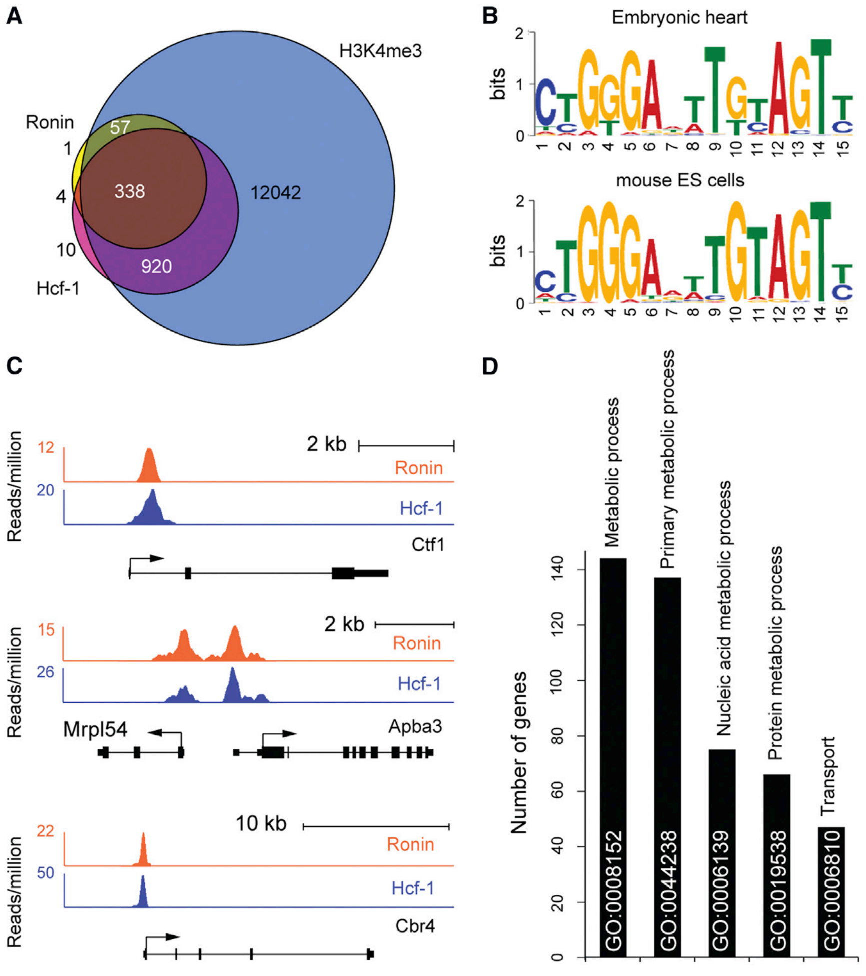

Figure 4. Ronin Cooperates with Hcf-1 to Regulate Expression of Metabolic Genes during Cardiac Development

(A) Venn diagram of ChIP-seq data representing embryonic hearts collected at E11.5. Most of the genes targeted by Ronin are also bound by Hcf-1 and are $H3K4me_3$ positive.

(B) Consensus sequences of the Ronin binding motif identified after ChIP-seq analysis are almost identical in embryonic heart (top) and embryonic stem cells (bottom).

(C) Examples of genes bound by both Ronin and Hcf-1 showing that signals are enriched immediately upstream of transcriptional start sites (arrow).

(D) PANTHER analysis classifying GO categories of all Ronin/Hcf-1 target genes assigns the vast majority of Ronin-bound genes to metabolic processes.

See also Table S1.

(2) all $H3K4me_3$-marked genes (Figures 6B and 6C; Table S3). *Nkx2.5*-driven Ronin loss significantly reduced the $H3K4me_3$ levels at Ronin/Hcf-1 target genes, such as those encoding Apba3 (amyloid beta [A4] precursor protein-binding, family A, member 3), Mrpl34 (mitochondrial ribosomal protein L34), Ctf1 (cardiotrophin 1), and Smarcal1 (SWI/SNF-related matrix-associated actin-dependent regulator of chromatin subfamily A-like protein 1) (Figure 6D), as confirmed by ChIP-qPCR (Figure 6E). Interestingly, $H3K4me_3$ levels at these genes were less affected overall after α*MHC* knockout. Together, our results are consistent with the hypothesis that Ronin and Hcf-1 are tethered to growth-critical gene promoters, potentially enhancing the number and stability of their $H3K4me_3$ marks (Wysocka et al., 2003). Alternatively, target gene malfunction might be caused by other as-yet-unidentified factors that decrease $H3K4me_3$. Thus, *Ronin* knockout may not only cause direct changes in $H3K4me_3$ levels and the expression of Ronin/Hcf-1 target genes, but may also affect genes indirectly by triggering the redistribution of additional Hcf-1 moieties to Ronin-independent targets. In this model, an excess of Hcf-1, unable to bind to Ronin target genes after knockout, might accumulate elsewhere in the genome, yielding ectopic $H3K4me_3$ deposits. Finally, it is likely that indirect mechanisms not dependent on the presence of Ronin or Hcf-1 are contributing at least in part to the increased $H3K4me_3$ levels at gene promoters with negative *Z* scores (Table S3) upon *Ronin* knockout.

DISCUSSION

Over the past two decades, numerous studies have identified most of the diverse progenitor cells that contribute to the proper formation of the developing heart (Ptaszek et al., 2012). However, the molecular cues that orchestrate this process remain under-investigated. Our group and others have shown that embryogenesis imposes stringent metabolic demands that are met not by a homeostatic system of housekeeping genes, but by a precisely regulated gene network, in which Ronin and other factors safeguard biomass assembly and other metabolic needs of embryonic stem cells (Dejosez et al., 2010; Wang et al., 2006).

Here, we asked whether Ronin contributes to mammalian cardiogenesis. Our results suggest that this regulatory factor controls cardiac growth during a critical midgestational period (about E9.5–E11.5), with Hcf-1 binding at active Ronin target genes and consequent changes in $H3K4me_3$ levels providing a potential mechanism of action. Support for this hypothesis comes from our observation that Ronin target genes were far more strongly dysregulated and $H3K4me_3$ levels at downregulated Ronin target genes more drastically decreased in early *Nkx2.5* knockout mice than in later α*MHC*-driven knockouts (Figure 6). This model explains both the lethal phenotype seen after the early knockout of *Ronin* and the relatively late onset of the α*MHC*-associated phenotype, which may reflect the slow buildup of pathogenic lesions. Indeed, the gene expression changes after α*MHC* knockout were both moderate and tolerable throughout embryonic development, perhaps because crucial chromatin marks such as $H3K4me_3$ are deposited at Ronin target genes before *Ronin* is removed by α*MHC*-Cre. Despite

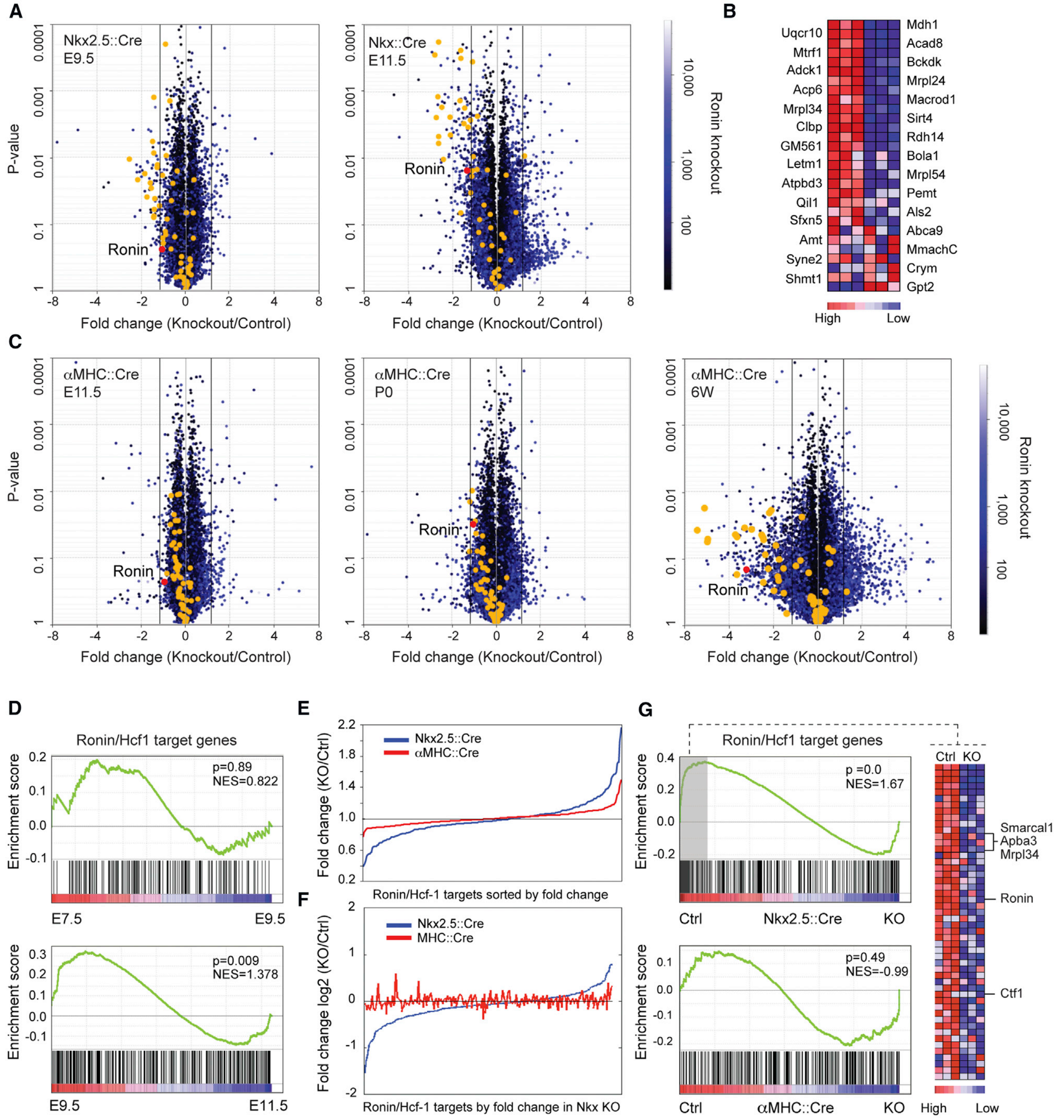

Figure 5. Ronin and Hcf-1 Cooperate to Regulate Expression of Target Genes during Early Cardiac Development to Control Cardiac Growth

(A) Volcano plots of microarray expression data obtained with RNA isolated from control or *Nkx2.5::Cre*-driven *Ronin* knockout heart tissue at the indicated time points. Yellow circles depict members of the mitochondrial gene signature that were discovered to respond to *Ronin* knockout in the retina (expression data with fold changes or p values outside the plot limits are not shown; n = 2 or 3 samples per group for the *αMHC*- or *Nkx2.5*-driven knockout hearts, respectively).

(B) Heatmap of mitochondrial genes highlighted in (A).

(C) Volcano plots of microarray expression data obtained with RNA isolated from control or *αMHC::Cre*-driven *Ronin* knockout heart tissue at indicated time points (n = 2 per group).

(D) GSEA shows that Ronin/Hcf-1 target genes are not significantly regulated between E7.5 and E9.5 (top), whereas significant changes are evident between E9.5 and E11.5 (bottom).

(legend continued on next page)

the lack of appreciable Ronin expression in adult cardiac myocytes, we identified persistent string-like subendocardial networks of Ronin-positive cells that might be part of the conductive system of the heart, providing another substrate for the late-onset phenotype. Hence, the postnatal cardiomyopathy and heart failure could be consequences of improper heart development (due to inadequate production, incorrect specification, or elimination of cardiomyocytes) or a later requirement for Ronin in heart function or perhaps a combination of both. Regardless of the mechanism of Ronin action, we recognize that the midgestational period of peak regulatory activity in our model coincides with genome-wide reprogramming of DNA methylation and histone modification in mammalian species (Feng et al., 2010). Because such changes are important for transcriptional gene silencing, they might well have contributed to the results reported here.

Unlike most transcription factors, which bind enhancers, Ronin almost exclusively prefers proximal promoters (Figure 4C) and lacks a discernible transactivation domain (Dejosez et al., 2010), suggesting that it has a distinct *modus operandi* for regulating transcription. The available evidence, including data from this study, does not indicate a clear link with any other heart development factor. Instead, studies on the prevalence and conservation of the Ronin binding motif (also known as M4 or LM4) have shown that it stands in striking isolation when compared with similarly conserved sequence elements, much like the CTCF-binding sequence (Xie et al., 2007). Subsequent studies have found that other DNA-binding factors, including ZNF143, GABP, YY1, and ICN1, can recognize part of the Ronin binding sequence, likely in a competitive fashion (Michaud et al., 2013), but the exact nature of these interactions is unclear and warrants further study. Finally, it will be interesting to determine whether Ronin's influence on genes involved in cardiogenesis uniformly requires Hcf-1 and methyltransferases or whether other regulatory mechanisms are involved.

Thus, in the developing heart, Ronin controls vital genetic programs across different cell types. This versatile regulatory role seems likely to offer advantages over the action of highly specialized, short-lived transcription factors, such as Nkx2.5 (Wamstad et al., 2012), in that it can buffer the activity of the short-lived regulators and assist with the transcriptional regulation of critical genes as cells progress through their developmental trajectories. Although the molecular functions of the THAP family proteins (including Ronin) have yet to be fully established, almost all of the characterized THAPs have been implicated in one or more human diseases, including heart disease (Balakrishnan et al., 2009), neurodegenerative conditions (Fuchs et al., 2009), congenital eye defects (Poché et al., 2016), craniofacial abnormalities (Achilleos et al., 2017a), inborn vitamin deficiencies (Quintana et al., 2017, Achilleos et al., 2017b), and various cancers (De Souza Santos et al., 2008; Johnson et al., 2010; Parker et al., 2012; Zhu et al., 2009). Given the lethal phenotypes observed in our mouse models, we predict that further analyses of human RONIN and its binding partners might reveal novel mutations that could underlie a spectrum of cardiomyopathies and perhaps open the door to new therapeutic strategies (Nakamura et al., 2012).

EXPERIMENTAL PROCEDURES

Mice

All experimental procedures and protocols of animal research were approved by the Institutional Animal Care and Use Committee of Baylor College of Medicine. *Nkx2.5::Cre* mice were provided by Dr. Robert Schwarz (Moses et al., 2001), *Tie2::Cre* mice were provided by Dr. Yanagisawa (Kisanuki et al., 2001), and *Rosa26tdRFP* mice were provided by Dr. Fehling (Luche et al., 2007). *αMHC::Cre*, *Ronin::lacZ*, and $Ronin^{fl/fl}$ mice were maintained as previously described (Agah et al., 1997; Dejosez et al., 2008, 2010). To create cardiac-specific Ronin conditional knockout animals, $Ronin^{fl/fl}$ mice were crossed with mice expressing the Cre recombinase under the control of a cardio-myocyte-specific promoter (*αMHC::Cre*, *Nkx2.5::Cre*, or *Tie2::Cre*) and backcrossed with $Ronin^{fl/fl}$ mice.

Tissue ChIP

Heart tissue from E11.5 embryos (at least 12 hearts pooled per group per sample) or P0 neonates (3 hearts pooled per group per sample) were collected and snap frozen in liquid nitrogen. After thawing, DNA and protein were crosslinked in 1% (v/v) formaldehyde-PBS for 15 min. The crosslinking reaction was quenched by the addition of glycine to a final concentration of 0.125 M and incubation for a further 5 min. All tissues were rinsed in cold PBS containing protease inhibitors (Roche) and PMSF before they were resuspended in 1 mL cold cell lysis buffer (10 mM Tris-HCl [pH 7.5], 10 mM NaCl, 3 mM $MgCl_2$, and 0.5% [v/v] NP-40) containing protease inhibitors and PMSF. The tissue was homogenized with a 2-mL dounce homogenizer and incubated on ice for 10 min to allow the release of nuclei. Nuclei were then collected by centrifugation at 3,000 rpm for 5 min, and each pellet was resuspended in cell lysis buffer and incubated for 5 min at 4°C with rotation. This step was repeated, followed by sonication (five times) with a Sonic dismembrator (Model 100, Fisher Scientific) for 6 s using a microtip probe (power output of 6). The tissues were next incubated with micrococcal nuclease (LS004798, Worthington Biochemical; 60 U/mL or 150 U/mL of E11.5 or P0 heart tissue, respectively). Insoluble debris was removed by centrifugation at 13,000 rpm for 10 min at 4°C, and supernatants were transferred to fresh reaction tubes. The extent of chromatin shearing was evaluated via 1.5% (w/v) agarose gel electrophoresis. The chromatin was precleared by the addition of 4 μL rabbit immunoglobulin G (IgG) or 6 μL preimmune serum and rotation for 1 hr at 4°C. 6 μL Ronin antiserum (G4275; Dejosez et al., 2008), 12 μL anti-Hcf-1 antibody (A301-400A, Bethyl), or 5 μL anti-trimethylated $H3K4me_3$ antibody (39159, Active Motif) were added to the supernatants, and samples were rotated overnight at 4°C. Salmon sperm DNA was added to a final concentration

(E) Comparison of microarray expression data from control or *Ronin* knockout heart tissue at E11.5 reveals a clear dysregulation of Ronin target genes after *Nkx2.5*-driven knockout (blue line), whereas *αMHC::Cre*-triggered *Ronin* deletion has a less pronounced effect (red line). Fold changes in the expression level of all Ronin/Hcf-1 target genes between knockouts and controls are shown.

(F) Sorting of the Ronin/Hcf-1 target genes by expression level in *Nkx2.5*-driven knockout shows that the genes dysregulated in *αMHC*-driven knockout hearts differ from those in *Nkx2.5*-driven knockouts. Fold changes (log2) between knockouts and controls are shown.

(G) GSEA reveals that *Nkx2.5*-driven knockout (top) significantly downregulates the expression of Ronin/Hcf-1 targets at E11.5. The heatmap (right) depicts the relative expression levels of the most affected genes. By contrast, *αMHC*-driven knockout (bottom) has no discernible effect on Ronin/Hcf-1 target gene distribution at E11.5. KO, knockout; Ctrl, control.

See also Figures S6–S9 and Table S2.

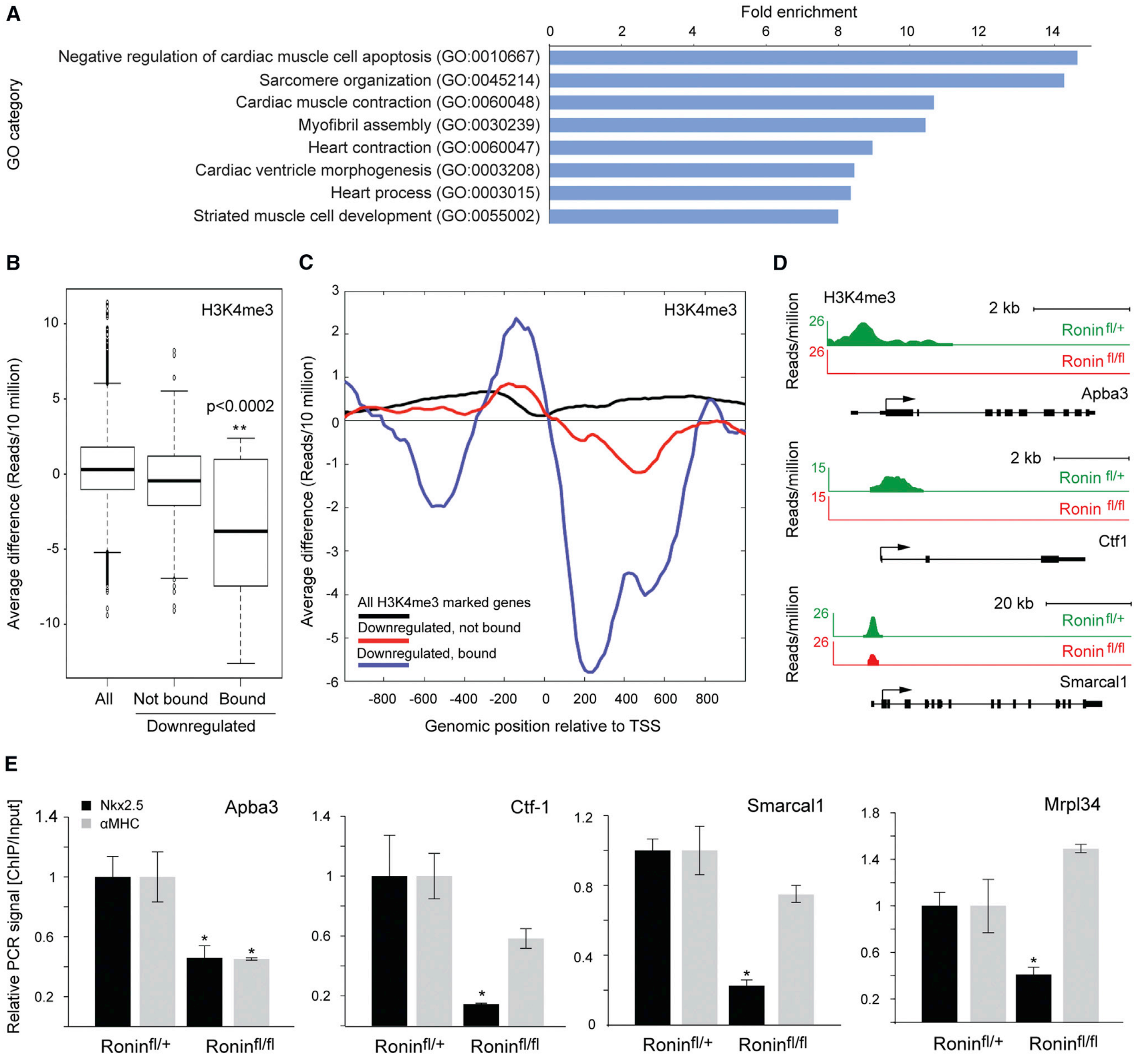

Figure 6. Ronin and Hcf-1 Control Target Genes during Early Cardiac Development by Modulating H3K4 Trimethylation

(A) ChIP-seq using $H3K4me_3$-specific antibodies and GO analysis of genes with significantly lower $H3K4me_3$ status than expected (*Z* score > 2) *in Nkx2.5::Cre*-driven *Ronin* knockout embryos at E11.5 show significant enrichment of categories related to cardiac function. Depicted are the top categories with fold enrichment scores of >8.

(B) Boxplot (median and interquantile values) of ChIP-seq signal differences comparing $H3K4me_3$ levels between *Nkx2.5*-knockout and control hearts at E11.5 (knockout minus control) at promoter regions (TSS to +500 bp) of all $H3K4me_3$-marked genes, downregulated Ronin target genes, or downregulated genes that are not bound by Ronin/Hcf-1. The $H3K4me_3$ signal of Ronin-regulated target genes is significantly reduced ($p < 0.0002$ by t test). TSS, transcriptional start site.

(C) The effect of Ronin loss on the spatial distribution of $H3K4me_3$ marks in the vicinity of relevant promoters, reported as the average difference in $H3K4me_3$ signal between knockout and control, according to the positions of Ronin target genes located ± 1 kb from the TSS. Black line, all $H3K4me_3$-marked genes; blue line, downregulated Ronin target genes; red line, all downregulated genes that are not bound by Ronin. Note reduction in $H3K4me_3$ upstream of the TSS as well as 200–400 kb downstream of the TSS.

(D) Examples of ChIP-seq signals at selected Ronin/Hcf-1 target genes show that the $H3K4me_3$ marks at Ronin target genes.

(E) ChIP-qPCR using an $H3K4me_3$-specific antibody demonstrates that the $H3K4me_3$ histone mark is greatly diminished in the promoter region of Ronin target genes in hearts isolated at E11.5 of *Nkx2.5::Cre* and at P0 of *αMHC::Cre* animals after knockout of *Ronin*. The PCR signal after ChIP is shown relative to the signal of the corresponding input sample (*$p < 0.05$ by t test, n = 3 per group). Mean ± SEM values are shown.

See also Table S3.

of 0.25 mg/mL, followed by incubation (with rotation) for 1 hr at 4°C. 50% Protein-A Sepharose (pre-treated by rotation with salmon sperm DNA at a final concentration of 0.25 mg/mL for 1 hr) was added to the samples, followed by incubation for 3 hr at 4°C, rotating. After four successive washes with 500 μL low-salt wash buffer (0.05% [w/v] SDS, 1% [v/v] Triton X-100, 2 mM EDTA, 20 mM Tris-HCl [pH 7.5], and 150 mM NaCl, with proteinase inhibitors), a high-salt wash buffer (0.05% [w/v] SDS, 1% [v/v] Triton X-100, 2 mM EDTA, 20 mM Tris-HCl [pH 7.5], and 500 mM NaCl, with proteinase inhibitors), LiCl wash buffer (0.25 M LiCl, 1% [v/v] NP-40, 1% [w/v] Na deoxycholate, 1 mM EDTA, and 10 mM Tris-HCl [pH 7.5], with proteinase inhibitors), and wash buffer (1% [v/v] Triton X-100, 2 mM EDTA, 20 mM Tris-HCl [pH 7.5], and 500 mM NaCl, with proteinase inhibitors), the Sepharose was resuspended in 300 μL Tris EDTA (TE) supplemented with 30 mM NaCl. Crosslinking was reversed by overnight incubation at 65° C, and the DNA was extracted twice with phenol/chloroform, precipitated with ethanol and resuspended in H_2O.

Microarray Analysis of Heart-Specific Ronin Conditional Knockout Mice

Hearts were isolated at E9.5 (10 hearts were pooled per group per replicate), E11.5, P0, or 6 weeks of age. RNA was isolated with the RNeasy kit (QIAGEN) following the manufacturer's standard protocol for isolation of RNA from animal tissue, including the on-column DNA digest. The microarray (MouseWG-6 v. 2.0 Illumina) was performed at the Microarray Core at Texas Children's Hospital. The resultant data were either normalized by quantile normalization (Figures 5D–5G; Table S2) with the Bioconductor package (Gentleman et al., 2004) or cubic spline normalization was performed with the Genepattern modules for Illumina expression file creation and normalization (Broad Institute). The E7.5 and E9.5 microarray datasets for the GSEA analysis shown in Figure 5D (top) were reconstructed from Masino et al., 2004.

Immunofluorescence

Embryos were fixed in 4% (v/v) paraformaldehyde at 4°C. Cryostat sections (8 μm each) were stained overnight at 4°C using specific antibodies diluted in PBS (anti-phospho-S6 ribosomal protein antibody [2211, Cell Signaling Technology, 1:200]), anti-RFP antibody (M155-3, MBL; 1:2,000), anti-α-Actinin antibody (A17811, Sigma; 1:400), or anti-phospho-H3 (Ser10) (06-570, Millipore; 1:100). Sections were washed three times with PBS, followed by incubation with the secondary antibodies conjugated to Alexa Fluor 488 or 594 (Invitrogen; 1:1,000). The sections were mounted with Vectashield mounting medium containing DAPI (Vector Laboratories) to visualize the nuclei. Slides were analyzed with an Axiocam 2 microscope (Carl Zeiss, Jena, Germany). Quantification after phospho-H3 (Ser10) staining was done with the ImageJ software. Five animals were analyzed per group. At least 3,600 myocardial (α-Actinin and DAPI positive) cells were counted per layer in each group.

DATA AND SOFTWARE AVAILABILITY

The accession number for the microarray data reported in this paper is GEO: GSE103959 (https://www.ncbi.nlm.nih.gov/geo/query/acc.cgi?acc=GSE103959).

SUPPLEMENTAL INFORMATION

Supplemental Information includes Supplemental Experimental Procedures, nine figures, and three tables and can be found with this article online at https://doi.org/10.1016/j.celrep.2017.10.036.

AUTHOR CONTRIBUTIONS

Conceptualization, T.P.Z., J.F., M.D.; Methodology, T.P.Z., J.F., M.D., P.G., A.L.B.; Formal Analysis, J.F., M.D., P.F., C.C.; Investigation, J.F.; Resources, M.S.; Writing – Original Draft, T.P.Z., M.D., J.F., M.D.S.; Writing – Review and Editing, T.P.Z., M.D., J.F.; Visualization, M.D., J.F.; Supervision, T.P.Z., M.D.; Project Administration, T.P.Z., M.D.; Funding Acquisition, T.P.Z.

ACKNOWLEDGMENTS

We thank D. Kearney at Texas Children's Hospital for electron microscopic analysis and the staff of the Baylor Mouse Phenotyping Core for gathering physiological data. We thank Drs. M. Dickinson and J. Martin for helpful discussions. We thank R. Schwarz for providing the *Nkx2.5::Cre* mice, M. Yanagisawa for the *Tie2::Cre* mice, and Hans-Jörg Fehling for the *Rosa26-tdRFP* mice. We also thank John Gilbert for critical reading of the manuscript and comments. This work was supported by the Cancer Prevention and Research Institute of Texas (RP170005), the Huffington Foundation, and NIH grants R01 GM077442-01 and P01 GM81627.

Received: May 20, 2017
Revised: September 1, 2017
Accepted: October 10, 2017
Published: November 7, 2017

REFERENCES

Achilleos, A., Tong, X., and Poché, R.A. (2017a). A new role of Ronin (Thap11) in the neural crest and craniofacial development in the mouse. FASEB J. *31* (*Suppl*), 387.2.

Achilleos, A., Tong, X., and Poché, R.A. (2017b). Ronin (Thap11) is implicated in a new cobalamin deficiency syndrome impacting the central nervous system. FASEB J. *31* (*Suppl*), 746.2.

Agah, R., Frenkel, P.A., French, B.A., Michael, L.H., Overbeek, P.A., and Schneider, M.D. (1997). Gene recombination in postmitotic cells. Targeted expression of Cre recombinase provokes cardiac-restricted, site-specific rearrangement in adult ventricular muscle in vivo. J. Clin. Invest. *100*, 169–179.

Balakrishnan, M.P., Cilenti, L., Mashak, Z., Popat, P., Alnemri, E.S., and Zervos, A.S. (2009). THAP5 is a human cardiac-specific inhibitor of cell cycle that is cleaved by the proapoptotic Omi/HtrA2 protease during cell death. Am. J. Physiol. Heart Circ. Physiol. *297*, H643–H653.

Chang, S., McKinsey, T.A., Zhang, C.L., Richardson, J.A., Hill, J.A., and Olson, E.N. (2004). Histone deacetylases 5 and 9 govern responsiveness of the heart to a subset of stress signals and play redundant roles in heart development. Mol. Cell. Biol. *24*, 8467–8476.

De Souza Santos, E., De Bessa, S.A., Netto, M.M., and Nagai, M.A. (2008). Silencing of LRRC49 and THAP10 genes by bidirectional promoter hypermethylation is a frequent event in breast cancer. Int. J. Oncol. *33*, 25–31.

Dejosez, M., Krumenacker, J.S., Zitur, L.J., Passeri, M., Chu, L.F., Songyang, Z., Thomson, J.A., and Zwaka, T.P. (2008). Ronin is essential for embryogenesis and the pluripotency of mouse embryonic stem cells. Cell *133*, 1162–1174.

Dejosez, M., Levine, S.S., Frampton, G.M., Whyte, W.A., Stratton, S.A., Barton, M.C., Gunaratne, P.H., Young, R.A., and Zwaka, T.P. (2010). Ronin/Hcf-1 binds to a hyperconserved enhancer element and regulates genes involved in the growth of embryonic stem cells. Genes Dev. *24*, 1479–1484.

Durruthy-Durruthy, J., Wossidlo, M., Pai, S., Takahashi, Y., Kang, G., Omberg, L., Chen, B., Nakauchi, H., Reijo Pera, R., and Sebastiano, V. (2016). Spatiotemporal reconstruction of the human blastocyst by single-cell gene-expression analysis informs induction of naive pluripotency. Dev. Cell *38*, 100–115.

Feng, S., Jacobsen, S.E., and Reik, W. (2010). Epigenetic reprogramming in plant and animal development. Science *330*, 622–627.

Fuchs, T., Gavarini, S., Saunders-Pullman, R., Raymond, D., Ehrlich, M.E., Bressman, S.B., and Ozelius, L.J. (2009). Mutations in the THAP1 gene are responsible for DYT6 primary torsion dystonia. Nat. Genet. *41*, 286–288.

Gentleman, R.C., Carey, V.J., Bates, D.M., Bolstad, B., Dettling, M., Dudoit, S., Ellis, B., Gautier, L., Ge, Y., Gentry, J., et al. (2004). Bioconductor: open software development for computational biology and bioinformatics. Genome Biol. *5*, R80.

Hiroi, Y., Kudoh, S., Monzen, K., Ikeda, Y., Yazaki, Y., Nagai, R., and Komuro, I. (2001). Tbx5 associates with Nkx2-5 and synergistically promotes cardiomyocyte differentiation. Nat. Genet. *28*, 276–280.

Hoffman, J.I., and Kaplan, S. (2002). The incidence of congenital heart disease. J. Am. Coll. Cardiol. *39*, 1890–1900.

Holz, M.K., Ballif, B.A., Gygi, S.P., and Blenis, J. (2005). mTOR and S6K1 mediate assembly of the translation preinitiation complex through dynamic protein interchange and ordered phosphorylation events. Cell *123*, 569–580.

Johnson, R.A., Wright, K.D., Poppleton, H., Mohankumar, K.M., Finkelstein, D., Pounds, S.B., Rand, V., Leary, S.E., White, E., Eden, C., et al. (2010). Cross-species genomics matches driver mutations and cell compartments to model ependymoma. Nature *466*, 632–636.

Kisanuki, Y.Y., Hammer, R.E., Miyazaki, J., Williams, S.C., Richardson, J.A., and Yanagisawa, M. (2001). Tie2-Cre transgenic mice: a new model for endothelial cell-lineage analysis in vivo. Dev. Biol. *230*, 230–242.

Kong, X.Z., Yin, R.H., Ning, H.M., Zheng, W.W., Dong, X.M., Yang, Y., Xu, F.F., Li, J.J., Zhan, Y.Q., Yu, M., et al. (2014). Effects of THAP11 on erythroid differentiation and megakaryocytic differentiation of K562 cells. PLoS One *9*, e91557.

Kuo, C.T., Morrisey, E.E., Anandappa, R., Sigrist, K., Lu, M.M., Parmacek, M.S., Soudais, C., and Leiden, J.M. (1997). GATA4 transcription factor is required for ventral morphogenesis and heart tube formation. Genes Dev. *11*, 1048–1060.

Li, G., Xu, A., Sim, S., Priest, J.R., Tian, X., Khan, T., Quertermous, T., Zhou, B., Tsao, P.S., Quake, S.R., et al. (2016). Transcriptomic profiling maps anatomically patterned subpopulations among single embryonic cardiac cells. Dev. Cell *39*, 491–507.

Lickert, H., Takeuchi, J.K., Von Both, I., Walls, J.R., McAuliffe, F., Adamson, S.L., Henkelman, R.M., Wrana, J.L., Rossant, J., and Bruneau, B.G. (2004). Baf60c is essential for function of BAF chromatin remodelling complexes in heart development. Nature *432*, 107–112.

Luche, H., Weber, O., Nageswara Rao, T., Blum, C., and Fehling, H.J. (2007). Faithful activation of an extra-bright red fluorescent protein in "knock-in" Cre-reporter mice ideally suited for lineage tracing studies. Eur. J. Immunol. *37*, 43–53.

Luxán, G., Casanova, J.C., Martínez-Poveda, B., Prados, B., D'Amato, G., MacGrogan, D., Gonzalez-Rajal, A., Dobarro, D., Torroja, C., Martinez, F., et al. (2013). Mutations in the NOTCH pathway regulator MIB1 cause left ventricular noncompaction cardiomyopathy. Nat. Med. *19*, 193–201.

Lyons, I., Parsons, L.M., Hartley, L., Li, R., Andrews, J.E., Robb, L., and Harvey, R.P. (1995). Myogenic and morphogenetic defects in the heart tubes of murine embryos lacking the homeo box gene Nkx2-5. Genes Dev. *9*, 1654–1666.

Ma, X.M., and Blenis, J. (2009). Molecular mechanisms of mTOR-mediated translational control. Nat. Rev. Mol. Cell Biol. *10*, 307–318.

Masino, A.M., Gallardo, T.D., Wilcox, C.A., Olson, E.N., Williams, R.S., and Garry, D.J. (2004). Transcriptional regulation of cardiac progenitor cell populations. Circ. Res. *95*, 389–397.

Michaud, J., Praz, V., James Faresse, N., Jnbaptiste, C.K., Tyagi, S., Schütz, F., and Herr, W. (2013). HCFC1 is a common component of active human CpG-island promoters and coincides with ZNF143, THAP11, YY1, and GABP transcription factor occupancy. Genome Res. *23*, 907–916.

Molkentin, J.D., Antos, C., Mercer, B., Taigen, T., Miano, J.M., and Olson, E.N. (2000). Direct activation of a GATA6 cardiac enhancer by Nkx2.5: evidence for a reinforcing regulatory network of Nkx2.5 and GATA transcription factors in the developing heart. Dev. Biol. *217*, 301–309.

Moses, K.A., DeMayo, F., Braun, R.M., Reecy, J.L., and Schwartz, R.J. (2001). Embryonic expression of an Nkx2-5/Cre gene using ROSA26 reporter mice. Genesis *31*, 176–180.

Nakamura, S., Yokota, D., Tan, L., Nagata, Y., Takemura, T., Hirano, I., Shigeno, K., Shibata, K., Fujisawa, S., and Ohnishi, K. (2012). Down-regulation of Thanatos-associated protein 11 by BCR-ABL promotes CML cell proliferation through c-Myc expression. Int. J. Cancer *130*, 1046–1059.

Olson, E.N. (2006). Gene regulatory networks in the evolution and development of the heart. Science *313*, 1922–1927.

Parker, J.B., Palchaudhuri, S., Yin, H., Wei, J., and Chakravarti, D. (2012). A transcriptional regulatory role of the THAP11-HCF-1 complex in colon cancer cell function. Mol. Cell. Biol. *32*, 1654–1670.

Poché, R.A., Zhang, M., Rueda, E.M., Tong, X., McElwee, M.L., Wong, L., Hsu, C.W., Dejosez, M., Burns, A.R., Fox, D.A., et al. (2016). RONIN is an essential transcriptional regulator of genes required for mitochondrial function in the developing retina. Cell Rep. *14*, 1684–1697.

Ptaszek, L.M., Mansour, M., Ruskin, J.N., and Chien, K.R. (2012). Towards regenerative therapy for cardiac disease. Lancet *379*, 933–942.

Quintana, A.M., Yu, H.C., Brebner, A., Pupavac, M., Geiger, E.A., Watson, A., Castro, V.L., Cheung, W., Chen, S.H., Watkins, D., et al. (2017). Mutations in THAP11 cause an inborn error of cobalamin metabolism and developmental abnormalities. Hum. Mol. Genet. *26*, 2838–2849.

Rosenthal, N.H., and Richard, P. (2010). Heart Development and Regeneration, First Edition (Academic Press).

Shikama, N., Lutz, W., Kretzschmar, R., Sauter, N., Roth, J.F., Marino, S., Wittwer, J., Scheidweiler, A., and Eckner, R. (2003). Essential function of p300 acetyltransferase activity in heart, lung and small intestine formation. EMBO J. *22*, 5175–5185.

Stanley, E.G., Biben, C., Elefanty, A., Barnett, L., Koentgen, F., Robb, L., and Harvey, R.P. (2002). Efficient Cre-mediated deletion in cardiac progenitor cells conferred by a 3'UTR-ires-Cre allele of the homeobox gene Nkx2-5. Int. J. Dev. Biol. *46*, 431–439.

Tyagi, S., Chabes, A.L., Wysocka, J., and Herr, W. (2007). E2F activation of S phase promoters via association with HCF-1 and the MLL family of histone H3K4 methyltransferases. Mol. Cell *27*, 107–119.

Wamstad, J.A., Alexander, J.M., Truty, R.M., Shrikumar, A., Li, F., Eilertson, K.E., Ding, H., Wylie, J.N., Pico, A.R., Capra, J.A., et al. (2012). Dynamic and coordinated epigenetic regulation of developmental transitions in the cardiac lineage. Cell *151*, 206–220.

Wang, J., Rao, S., Chu, J., Shen, X., Levasseur, D.N., Theunissen, T.W., and Orkin, S.H. (2006). A protein interaction network for pluripotency of embryonic stem cells. Nature *444*, 364–368.

Wysocka, J., Myers, M.P., Laherty, C.D., Eisenman, R.N., and Herr, W. (2003). Human Sin3 deacetylase and trithorax-related Set1/Ash2 histone H3-K4 methyltransferase are tethered together selectively by the cell-proliferation factor HCF-1. Genes Dev. *17*, 896–911.

Xie, X., Mikkelsen, T.S., Gnirke, A., Lindblad-Toh, K., Kellis, M., and Lander, E.S. (2007). Systematic discovery of regulatory motifs in conserved regions of the human genome, including thousands of CTCF insulator sites. Proc. Natl. Acad. Sci. USA *104*, 7145–7150.

Zhu, C.Y., Li, C.Y., Li, Y., Zhan, Y.Q., Li, Y.H., Xu, C.W., Xu, W.X., Sun, H.B., and Yang, X.M. (2009). Cell growth suppression by thanatos-associated protein 11(THAP11) is mediated by transcriptional downregulation of c-Myc. Cell Death Differ. *16*, 395–405.

Cell Reports, Volume *21*

Supplemental Information

Ronin Governs Early Heart Development

by Controlling Core Gene Expression Programs

Jun Fujita, Pablo Freire, Cristian Coarfa, Ashley L. Benham, Preethi Gunaratne, Michael D. Schneider, Marion Dejosez, and Thomas P. Zwaka

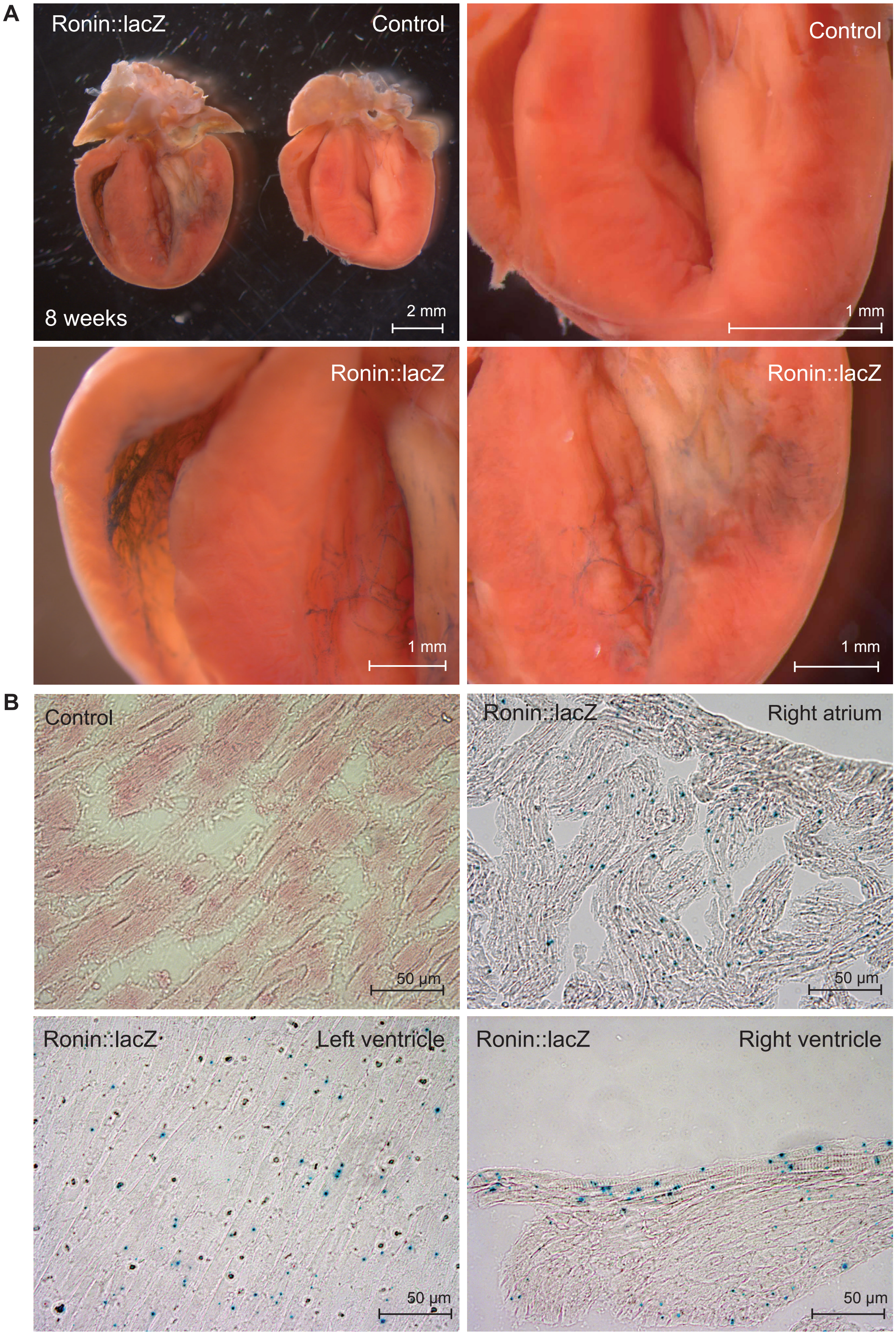

Figure S1: LacZ staining of *Ronin::lacZ* and control animals at 8 weeks of age. Related to Figure 1. (**A**) Images of entire hearts cut sagitally along the midsection. (**B**) Images of heart sections.

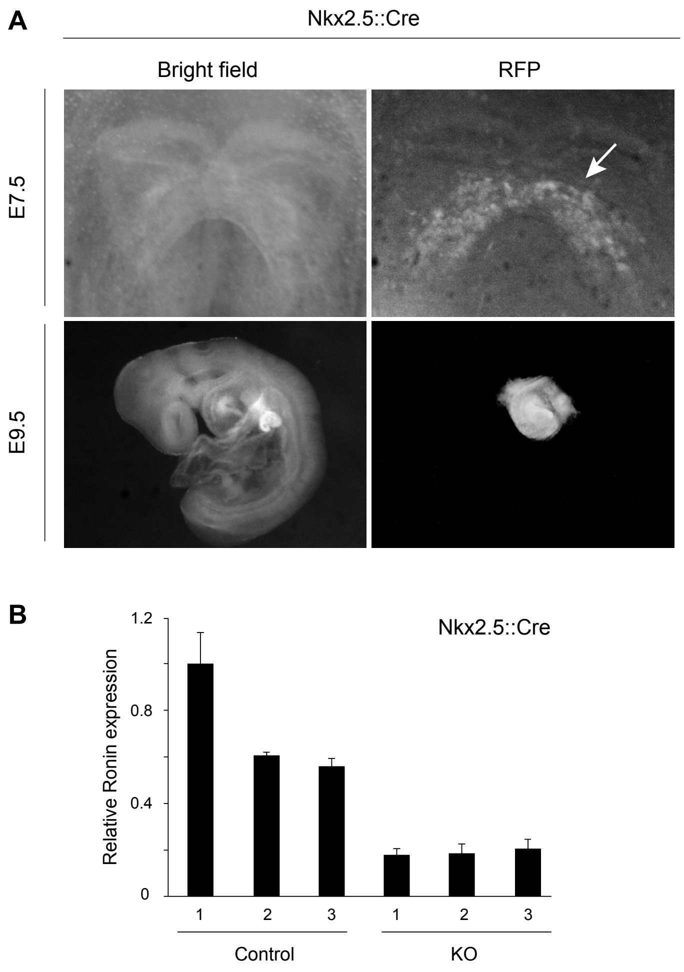

C

Ronin	Nkx2.5::Cre +/o	Nkx2.5::Cre o/o	αMHC::Cre +/o	αMHC::Cre o/o
fl/+	19	18	22	24
fl/fl	0	25	22	21

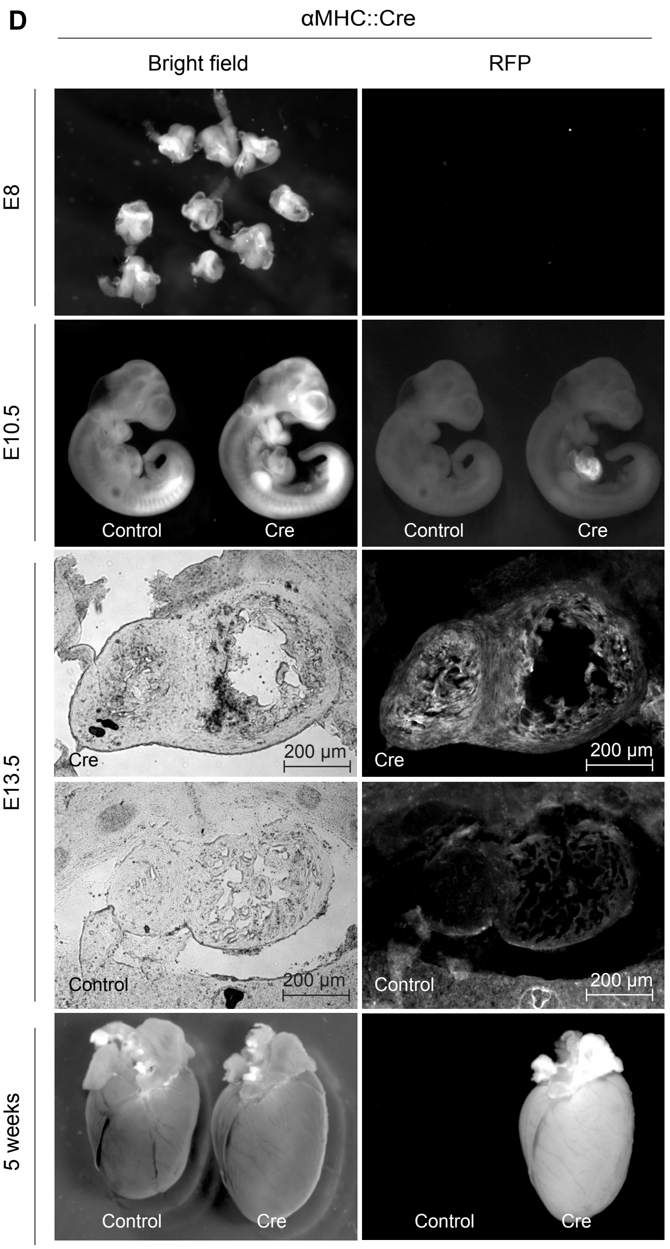

Figure S2. Validation of the temporal and spatial *Nkx.2.5*- and *αMHC*-driven Cre expression. Related to Figures 2 and 3. *Nkx2.5*::*Cre* or *αMHC*::*Cre* animals were crossed with *Rosa26*$^{tdRFP/tdRFP}$ reporter animals, and RFP-positive cells were analyzed at various developmental stages. (**A**) *Nkx2.5::Cre*: RFP-positive cells emerge first in the cardiac crescent (white arrow) at E7.5, and then appear throughout the heart at E9.5. This supports the notion that *Nkx2.5*::Cre activity closely mirrors endogenous *Nkx2.5* expression and thus that the *Ronin* gene is excised at E7.5. (**B**) RT-PCR of *Ronin* at E11.5 confirmed significant reduction of Ronin expression after knockout (n=3; p=0.0187 by t-test). (**C**) Distribution of *Ronin* alleles in crosses with *Nkx2.5*::Cre and *αMHC*::Cre animals at P0 as indicated. No viable embryos were identified after *Nkx2.5*-driven knockout, whereas *αMHC*- knockout showed typical mendelian ratios. (**D**) *αMHC::Cre*: While no Cre activity is detected at E8.0, and from E10.5 on, the bulk of the heart is RFP-positive (>90% of all cardiac cells), suggesting *αMHC::Cre*-mediated conditional deletion of *Ronin* as early as E10.5 that is sustained thereafter, as seen in sections at E13.5 or at 5 weeks of age.

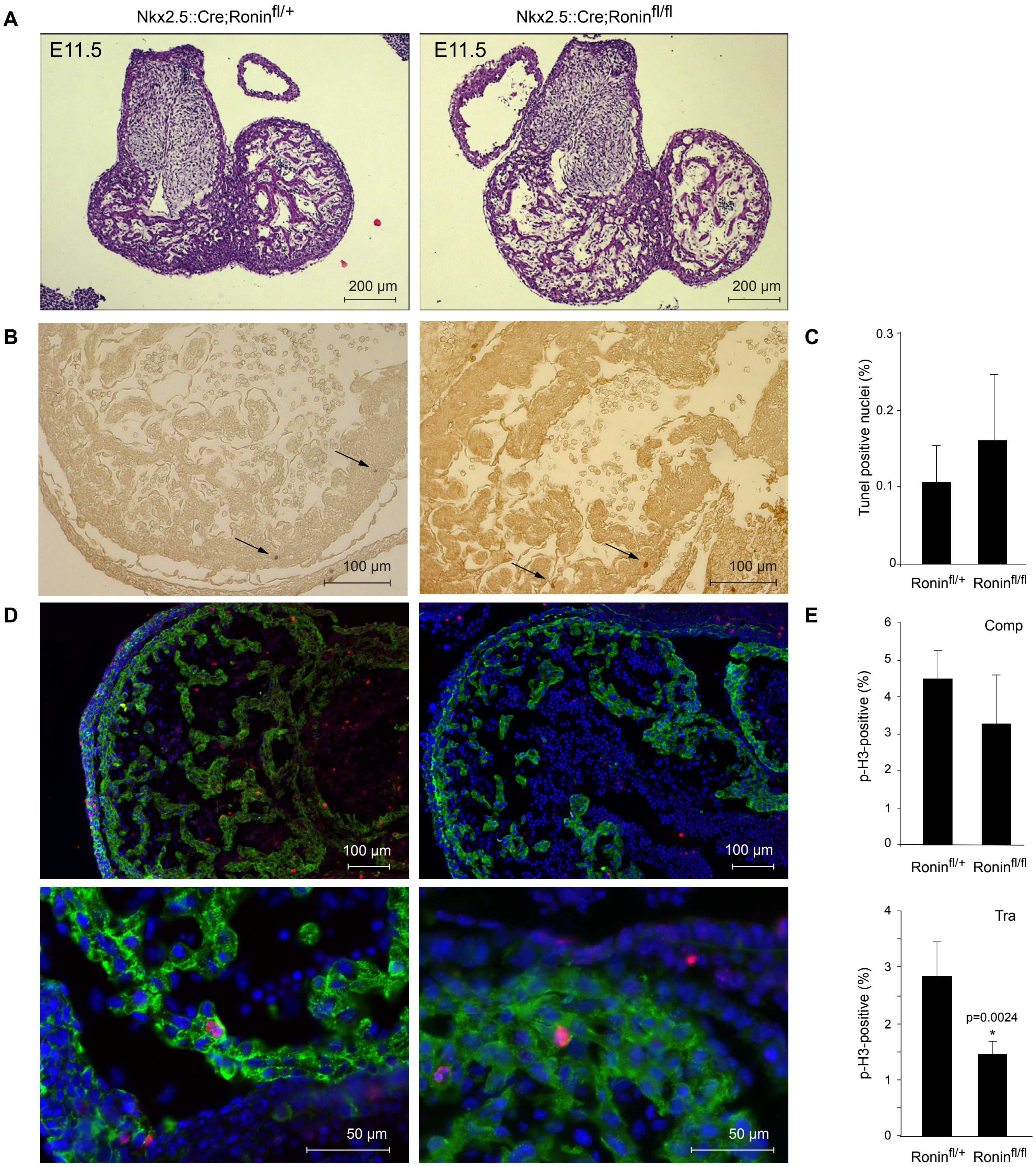

Figure S3. *Nkx2.5*-driven loss of Ronin leads to cardiac defects and is associated with cell death and attenuated cell proliferation by E11.5. Related to Figure 2. (**A**) Heart sections after H&E staining show that the heart chamber walls of *Nkx2.5*-driven *Ronin* knockout mice (right) are much thinner than those of heterozygous controls (left) at E11.5. (**B**) Sections of E11.5 heart tissue after tunel-staining to detect apoptotic cell death. (**C**) Quantification of experiment shown in (B) indicates an increase, albeit not statistically significant, in programmed cell death in *Nkx2.5*-driven *Ronin* knockout heart tissue. (**D**) Phospho-Histone 3 (p-H3) immunostaining of E11.5 heart tissue sections shows a decrease of mitotic cells in *Nkx2.5*-driven *Ronin* knockout animals when compared with heterozygous controls in the free ventricular walls (excluding atrium and septum). Red, p-H3; green, α–Actinin; blue, Dapi. (**E**) Quantification of experiment shown in (D) confirms a decrease of mitotic cells in both myocardial layers with a statistically significant difference in the trabecular layer. Comp, compact layer; Tra, trabecular layer. Five animals per group were analyzed and, at least 3600 nuclei were counted per layer.

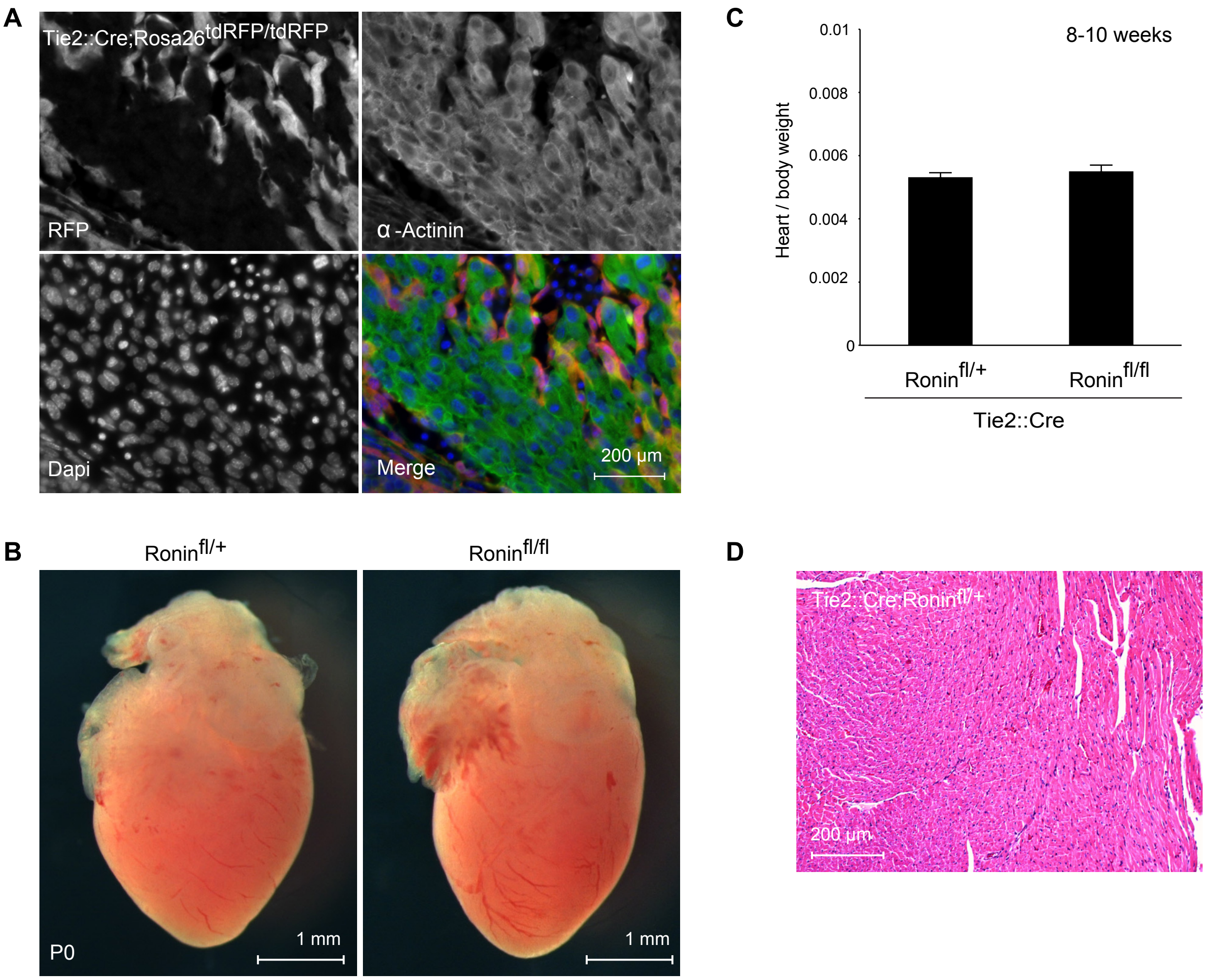

Figure S4. Characterization of *Tie2*::Cre driven *Ronin* knockout mice. Related to Figure 2. (**A**) *Tie2*::Cre animals were crossed with *Rosa26*$^{tdRFP/tdRFP}$ reporter animals. Immunofluorescence analysis of RFP-positive cells in heart tissue sections at E13.5 shows RFP signal exclusively in the endocardium, and not in the adjacent (α-Actinin positive) muscle. (**B**) At P0 (top) there are no overt macroscopic differences between hearts isolated from *Tie2*-driven *Ronin* knockout and those of heterozygous control animals. (**C**) The heart-to-body weight ratio in knockout and heterozygous controls shows no significant differences. (**D**) H&E staining of tissue sections obtained from heterozygous animals at 8 weeks of age shows well-ordered alignment of normal cardiomyocytes.

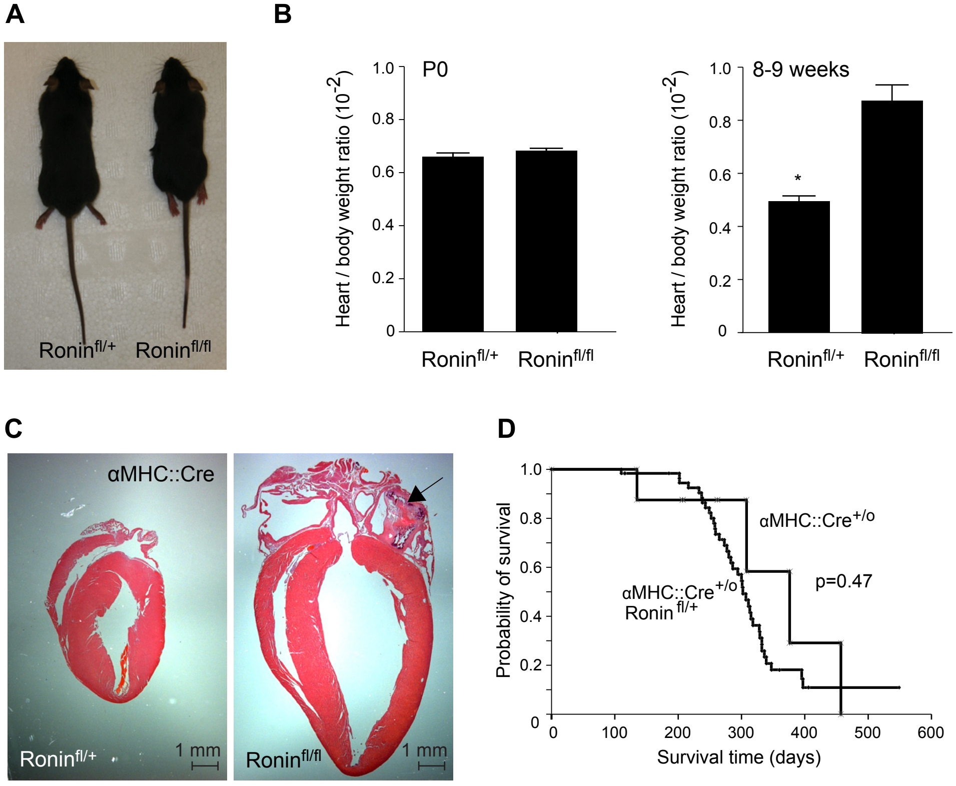

Figure S5. Phenotypic analysis of hearts isolated from *αMHC*-driven *Ronin* knockout animals at 12 weeks of age. Related to Figure 3. (**A**) Male knockout animals are significantly smaller than their heterozygous counterparts (26.6 g vs. 28.1 g; p=0.029 by t-test), possibly due to cardiac cachexia. (**B**) The heart-to-body weight ratio was identical in *Ronin* knockout animals and controls at P0 (left), but significantly higher in *Ronin* knockout animals by the age of 8-9 weeks (right). *p<0.05 by t-test. (**C**) Macroscopic histology of the heart of knockout animals at 12 weeks of age shows that all four chambers are dilated. Additionally, a very large thrombus (arrow) is apparent in the left atrium in comparison to heterozygous controls. (**D**) Kaplan-Meier analysis shows that the survival times of *αMHC*-driven heterozygous *Ronin* knockout animals do not differ significantly from *αMHC::Cre*-expressing animals. p-value was determined by log-rank test.

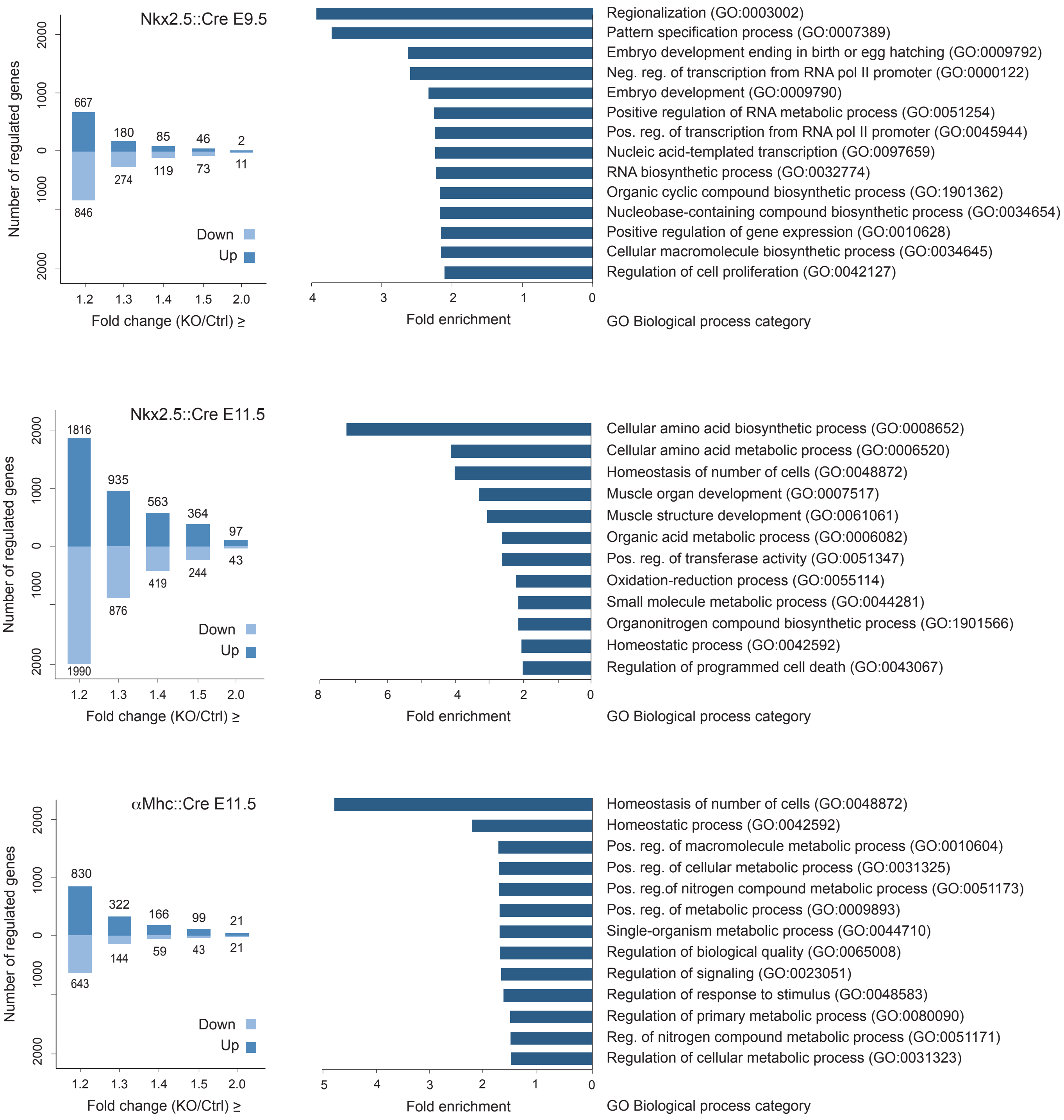

Figure S6. Analysis of differentially expressed genes in *Nkx2.5*- or *αMHC*-driven Ronin knockout compared with control heart tissue during embryonic development at E9.5 or E11.5. Related to Figure 5. Shown are the number of up- or downregulated genes (left) and selected phenotypically relevant gene ontology categories of biological processes (right), that are significantly enriched (with a p-value lower or equal to 0.5) using gene sets with a cutoff where at least 300 genes are present (1.3-, 1.5- or 1.3-fold, respectively, from top to bottom). Ctrl, control; KO, knockout; GO, gene ontology.

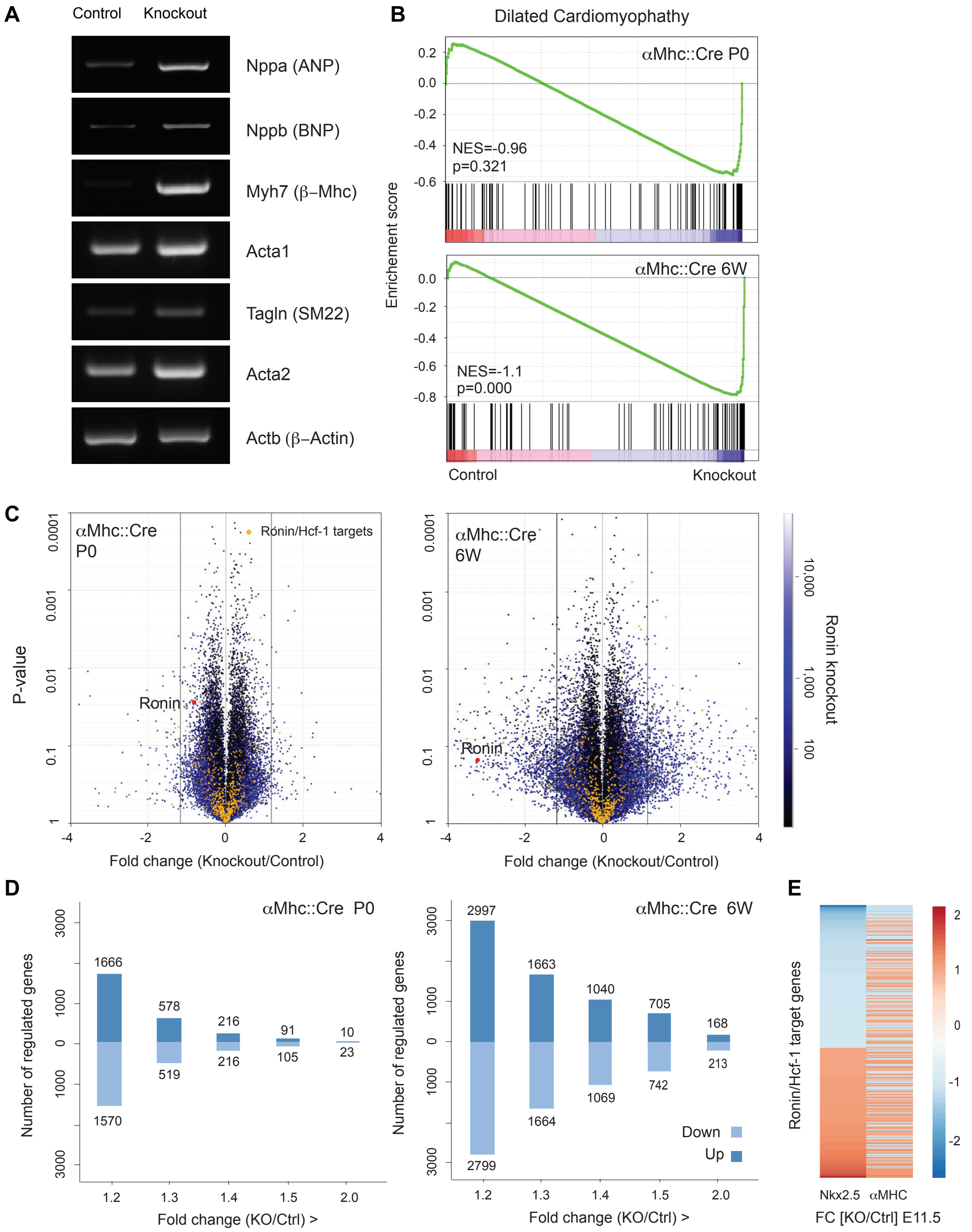

Figure S7. Gene expression analysis of hearts isolated from *Ronin* knockout animals. Related to Figure 5. (**A**) RT-PCR shows that selected markers of dilated cardiomyopathy are upregulated in heart tissue of *Ronin* knockout animals at 6 weeks of age. (**B**) GSEA analysis reveals the dysregulation of a gene set related to dilative cardiomyopathy at P0 after α*MHC*-driven *Ronin* knockout that becomes significantly enriched at 6 weeks of age. (**C**) Volcano plots of gene expression data obtained from microarray analyses at indicated time points. Yellow circles, Ronin/Hcf-1 targets. (**D**) Number of up- or downregulated genes in heart tissue after *Ronin* knockout when compared with controls. (**E**) Heatmap of Ronin/Hcf-1 target gene expression in heart tissue after *Ronin* knockout at E11.5. Target genes are sorted by fold change in *Nkx2.5*-knockout. FC, Fold change; NES, normalized enrichment score. Ctrl, control; KO, knockout; 6W, 6 weeks.

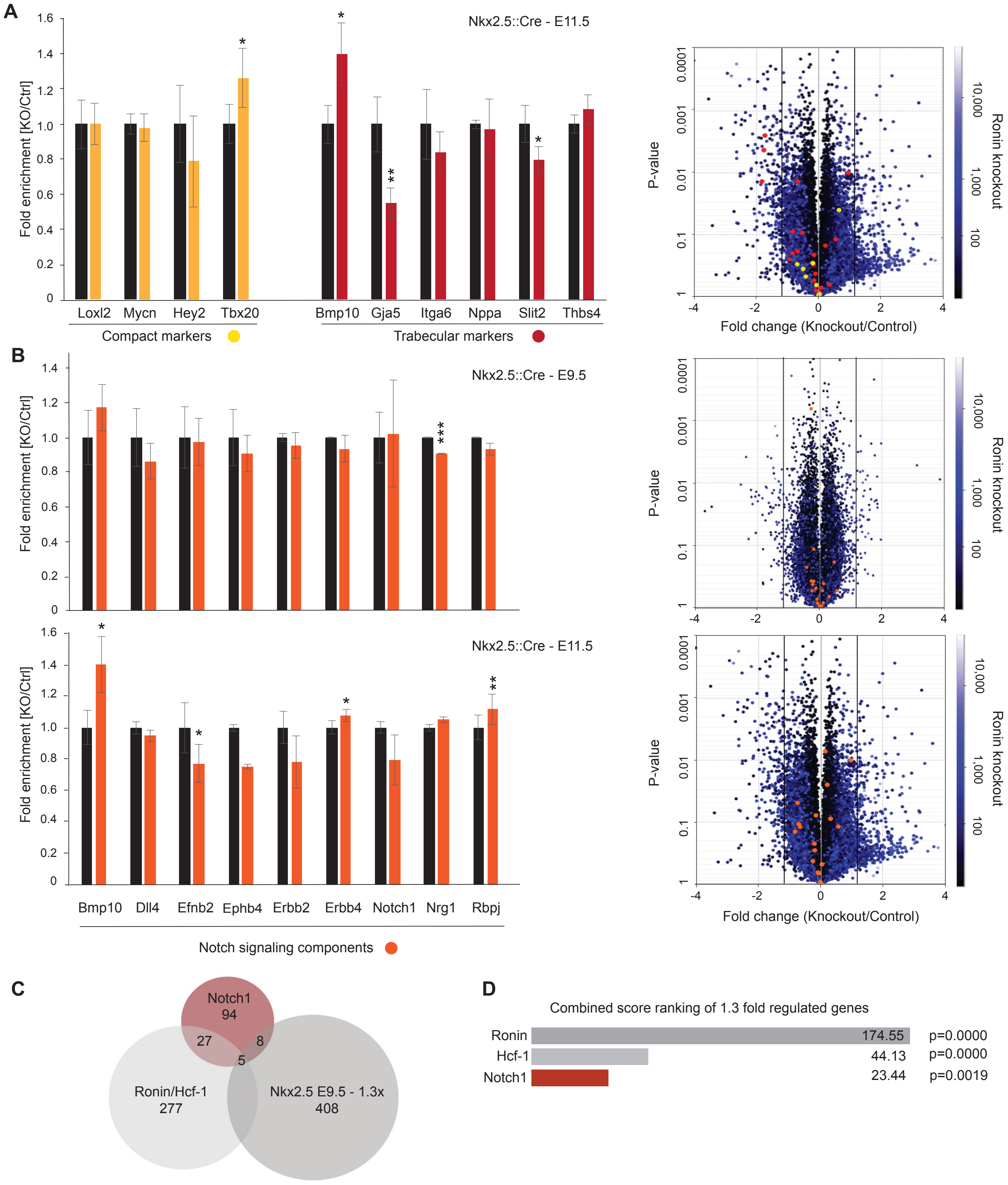

Figure S8. Differential gene expression analysis of selected gene sets at E9.5 and E11.5 of *Nkx2.5*-driven *Ronin* knockout heart tissue. Related to Figure 5. (**A**) Markers specific for the myocardial compact or trabecular layers show mild changes in both layers at E11.5 after *Ronin* knockout (left). The gene probes are highlighted within the volcano plot (left) relative to all other gene probes detected in microarrays at E11.5. (**B**) Analyses of Notch signaling components do not reveal a specific direct involvement in the phenotypic changes observed after *Ronin* knockout at E9.5 (top) or E11.5 (bottom). Gene expression is illustrated as described in (A). (**C**) Venn diagram showing the overlap of Ronin/Hcf-1 with Notch1 targets (Chip enrichment analysis (ChEA) 2016 database set *NOTCH1_17114293_ChIP-ChIP_T-ALL_Human*) and the genes that are more than 1.3-fold up- or downregulated at E9.5. after *Nkx2.5*-driven *Ronin* knockout. (**D**) ChEA of genes that are up- or downregulated more than 1.3-fold in heart tissue at E9.5 after *Nkx2.5*-knockout. Shown are the combined score and p-value for each category. Ronin, ChEA 2016 database set: Thap11_20581084_ChIP-Seq_MESCs_Mouse; ChEA 2016 database set Hcf-1, HCFC1_20581084_-ChIP-Seq_MESCs_Mouse; Notch1, ChEA 2016 database set as in (C). *p<0.05; **p<0.01; ***p<0.001.

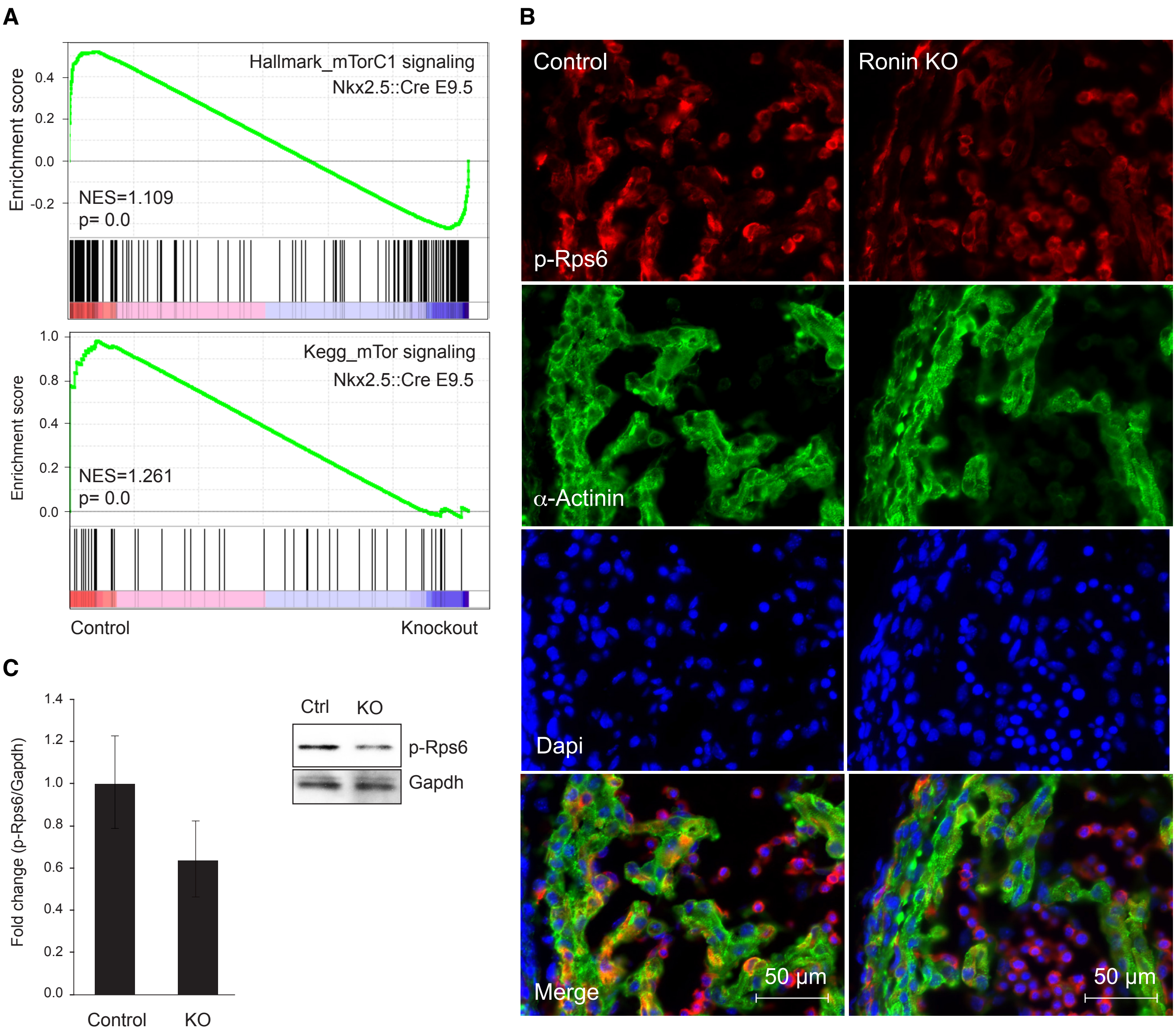

Figure S9. mTor activity is reduced in *Nkx2.5*-driven *Ronin* knockout animals at E11.5. Related to Figure 5. (**A**) Gene set enrichment analysis (GSEA) shows significant enrichment of the “Hallmark” and “Kegg” mTor signaling components in control tissue after *Nkx2.5::Cre*-driven knockout of *Ronin*. (**B**) Detection of the phosphorylation levels of the mTor target Rps6 at E11.5 by immunofluorescence reveals that heart tissue of *Nkx2.5*-knockout animals has lower Rps6 phosphorylation levels than heterozygous controls while α-Actinin or Gapdh remain at similar protein levels. Red, p-Rps6; green, α–Actinin; blue, Dapi. (**C**) Quantification of Western blot analysis confirms decreased phosphorylation of p-Rps6 upon *Ronin* knockout (n=3 ± SEM).

SUPPLEMENTAL EXPERIMENTAL PROCEDURES

Genotyping

The genotypes of all offspring were analyzed with DNA isolated from either the yolk sac of embryos or adult tail biopsies. The 5` and 3` primers amplifying *Nkx2.5::Cre*, *αMHC::Cre, Rosa25-tdRFP*, *Tie2::Cre* and the *Ronin*^{fl} alleles were: 5'-GATTA GCTTA AGCGG AGCTG GGTGT CC-3', 5'-GTTCT GGAAC CAGAT CTTGA CCTGC GTGGA-3'; 5'-GCCGC ATAAC CAGTG AAACA GCATT GC-3', 5'-ATGAC AGACA GAT CC CTC CT ATCTC C-3'; 5'-CTCAT CACTC GTTGC ATCAT CGAC-3', 5'-AAGAC CGCGA AGAGT TTGTC C-3'; 5'-T AAGC CTGCC CAGAA GACTC C-3', 5'-AAGGG AGCTG CAGTG GAGTA -3'; 5`-GCGGT CTGGC AGTAA AAACT ATC-3', 5'-GTGAA ACAGC ATTGC TGTCA CTT-3'; 5'-TACCC AGAGC GCTTG CGCTC ACC AG-3', 5'-TCCA G ATGAA GCTCG TCCTA AGCGA-3', respectively.

Hematoxylin-and-Eosin Staining

Adult heart and embryonic tissues were fixed in 10% (v/v) formalin and transferred to 70% (v/v) ethanol. The tissue was embedded in paraffin and 10-µm sections were stained with hematoxylin and eosin by the Histology Service of the Department of Pathology at Baylor College of Medicine.

X-gal Staining

Embryos and heart tissues were fixed in fixation buffer (0.2% [w/v] glutaraldehyde, 2 mM $MgCl_2$, and 5 mM EGTA) for 15 min, followed by three 15 min washes in detergent-containing buffer (2 mM $MgCl_2$, 0.01% [w/v] sodium deoxycholate, and 0.02% [v/v] NP-40). The tissue was then stained with X-gal solution (40 mg/ml X-gal in N,N, dimethylformamide mixed 1:40 with X-gal reaction buffer (100 µl 1M $MgCl_2$, 49.4 ml PBS, 82 mg potassium ferricyanide and 106 mg potassium ferrocyanide) for 4 – 5 hrs. They were washed three times in PBS, fixed in 4 % (v/v) paraformaldehyde overnight, and preserved in 70 % EtOH.

Electron Microscopy

Hearts were excised, fixed in glutaraldehyde, sectioned, and examined at the Department of Pathology at the Texas Children's Hospital.

Electrocardiography and Echocardiography

Electrocardiography (ECG) and echocardiography recordings were performed in the Mouse Phenotyping Core Facility at Baylor College of Medicine. ECG recordings were made with ECGenie, which provides a lead II ECG from the paws in conscious mice. Echocardiography was performed with a Visualsonic Vevo 770 Imaging System. Mice were anesthetized with isoflurane to maintain spontaneous breathing. The left ventricular ejection fraction and left ventricular internal end-diastolic diameter were measured.

ChIP-sequencing and ChIP-qPCR

Adapter ligation and ChIP-sequencing was performed as described previously (Dejosez et al., 2010). The ChIP-derived reads were aligned to the mouse genome (NCBI build 37, UCSC build mm9) using the FindPeaks software (UBC). Chip sequencing data were validated by ChIP-qPCR using Sybr Green in real time PCR analysis. The primers amplifying the *Apba3*, *Ctf-1*, *Mrpl34* and *Smarcal1* promoters were: 5'-TGAGT TTCAA GGGTC AGAGC -3', 5'-AACTC AGGCT TTAGG TCGTG -3'; 5'-AAAGC TGAAA GACTG GAGGG -3', 5'-TCCGA AGCCT GTATT TTGAG C-3'; 5'-GTGAG TGTTC AGAGA TACCC TG-3', 5'-TGTAT GACTT TCTGA GCCGC -3'; 5'-GCTAG GTAAG AAAGT GGGCT G-3', 5'-TCCTC TTGCC GTATT TGGTC -3', respectively. All samples were tested in triplicate and data were normalized to PCR signals of the corresponding input samples.

Bioinformatics Analyses

The logo reflecting the Ronin binding motif was created with Weblogo software (http://weblogo.berkeley .edu/). The Venn diagram was generated with the Venn Charts Google Developers online tool (https://developers.google.com). GSEA was conducted with GenePattern (Broad Institute; Reich et al., 2006). The PANTHER tool (Thomas et al., 2003) was implemented to determine functional categories of the Ronin/Hcf-1 target genes or the genes that were differentially expressed after *Ronin* knockout. The chromatin enrichment analysis (ChEA) was performed with the web-based Enrichr tool (Kuleshov et al., 2016). The heat map in Figure S7 was produced with the ClustVis webtool (targets were sorted by fold change. The $H3K4me_3$ status between different gene sets was compared with the Genboree signal comparison tool (Bioinformatics Research Laboratory, Baylor College of Medicine; www.genboree.org). We used a linear regression of the $H3K4me_3$ signals of control and *Nkx2.5*-driven Ronin knockout signals over all gene promoters (Table S6). The results are annotated by z-scores. Positive z-scores indicate promoters with lower $H3K4me_3$ levels, negative z-scores indicate promoters with higher $H3K4me_3$ levels in *Ronin* knockout tissue by comparison with controls. Z-scores above 2 were considered significant.

Western Blot Analysis

Western blot analysis was performed as previously described (Dejosez et al., 2008). Primary antibodies were anti-phospho-S6 (2211, Cell Signaling; 1:1000) and anti-GAPDH (ab9485, Abcam; 1:2500).

Tunel Staining

Tunel staining was performed with the DeadEnd™ Colorimetric TUNEL System (Promega) in accordance to the manufacturers protocol.

Statistics

All values are presented as means ± SEM or SD as indicated. Statistical significance was evaluated with the unpaired Student's t-test for comparison of two means. The Kaplan-Meier method was used to estimate survival rates, while mortality was computed with the χ^2 test. A p-value of < 0.05 was considered to indicate significance.

SUPPLEMENTAL REFERENCES

Kuleshov, M.V., Jones, M.R., Rouillard, A.D., Fernandez, N.F., Duan, Q., Wang, Z., Koplev, S., Jenkins, S.L., Jagodnik, K.M., Lachmann, A., *et al.* (2016). Enrichr: a comprehensive gene set enrichment analysis web server 2016 update. Nucleic Acids Res *44* :W90-97.

Reich M., Liefeld, T., Gould, J., Lerner, J., Tamayo, P., Mesirov, J.P. (2006). GenePattern 2.0. Nat Genet *38*, 500-501.

Thomas, P.D., Kejariwal, A., Campbell, M.J., Mi, H., Diemer, K., Guo, N., Ladunga, I., Ulitsky-Lazareva, B., Muruganujan, A., Rabkin, S.*, et al.* (2003). PANTHER: a browsable database of gene products organized by biological function, using curated protein family and subfamily classification. Nucleic Acids Res *31*, 334-341.
